# DEM modelling of railway ballast using the Conical Damage Model: a comprehensive parametrisation strategy

**DOI:** 10.1007/s10035-021-01198-z

**Published:** 2022-01-24

**Authors:** Bettina Suhr, William A. Skipper, Roger Lewis, Klaus Six

**Affiliations:** 1 Virtual Vehicle Research GmbH, Inffeldgasse 21/A, 8010 Graz, Austria; 2 Department of Mechanical Engineering, The University of Sheffield, Mappin Street, Sheffield, S1 3JD UK

**Keywords:** Railway ballast, DEM modelling, Contact modelling, Calibration, Parameter ambiguity

## Abstract

Despite ongoing research, the parametrisation of a DEM model is a challenging task, as it depends strongly on the particle shape representation used, particle-particle contact law and the simulated applications: for railway ballast e.g. lab tests or track conditions. The authors previously modelled railway ballast with a DEM model using a simple particle shape. The DEM model was parametrised, by trial-and-error, to compression and direct shear test results. A good agreement between DEM model and experimental results was achieved only when the Conical Damage Model (CDM) was used as the contact law. Compared to the well-known linear-spring Cundall-Strack law or the Hertz-Mindlin law, this contact law takes into account additional physical effects (e.g. edge breakage) occurring in the experiment. Little is known on the influence of the CDM model parameters on the simulation results or on possible parameter ambiguities. This lack of knowledge hinders a reliable and efficient parametrisation of DEM models using different particle shapes. Both points are addressed in this work in detail by investigating a DEM model for railway ballast using one simple particle shape. Suggestions for a parametrisation strategy of reduced computational effort are formulated and tested using a second particle shape. In future works, the newly presented parametrisation strategy can help to calibrate different DEM models and to study the influence of particle shape.

## Introduction

The Discrete Element Method (DEM) is a frequently used method for the simulation of granular materials. To build a reliable DEM model, three modelling steps are necessary: representation of particle geometry (shape and size), choice of an appropriate contact law (taking into account all necessary physical mechanisms) and a thorough calibration of the contact law’s parameters. The DEM modelling of railway ballast is an active field of research regarding these three steps.

*Particle shape* is addressed in many different studies in the literature. Rigid clumps of spheres are constructed from 2D or 3D scanner data of ballast stones e.g. in [10–12, 17, 24, 34]. The constructed clumps are detailed shape models, which usually consist of a high number of spheres (above 10 in [17] and above 50 in [11]). The computational effort for such high detail shape models increases not only with the number of spheres, but also with decreasing sphere radii, needed to construct sharp corners or edges. For the DEM simulation of railway ballast polyhedra are also used in the literature. In approaches used by [14, 16, 27, 47], data from 3D-scanned ballast stones can be used to build polyhedral DEM particles, with respect to certain shape descriptors. The computational effort of using polyhedra is in general higher than that of clumps of spheres (dependent on the actual shape and the simulation software used) and increases with the number of corners being modelled. In [1, 13], potential particles for the simulation of triaxial tests of railway ballast are used. [1] present a method to manually adapt the shape of a potential particle to the shape of a ballast stone. Independent from the particle type chosen, most citations mentioned above used relatively complex shapes to model the complex shape of railway ballast stones in high detail. Exceptions, e.g. [5, 6, 21], who used simple shape models, only obtained qualitative, not quantitative agreement between simulations and experiments.

*Contact modelling* is addressed in the literature less frequently than particle shape modelling. Many of the studies cited above applied the linear spring model of Cundall and Strack, [8], or the simplified Hertz-Mindlin model, see [46]. Modelling of particle breakage can be achieved using clumps of bonded spheres and corresponding contact laws, see e.g. [18, 22]. Recently, a new contact model was developed in [9] taking abrasion of railway ballast into account. In [13], Harkness et al. conducted simulations of monotone and cyclic triaxial tests on railway ballast using potential particles. They found that the Hertz-Mindlin model could not be parametrised to give both low initial stiffness in monotonic loading at high confining pressures and high elastic stiffness in cyclic loading. To overcome this problem, they introduced a modified contact model to account for additional physical phenomena occurring in the test: the Conical Damage Model (CDM). In the CDM law the elastic part of the material behaviour is modelled via the Hertz law. Additionally, a kind of ideal plasticity is is introduced to model damage at a contact (e.g. to take into account edge breakage). The CDM law has four parameters (two more than the classical Hertz-Mindlin law).

*Parametrisation* strategies are usually addressed in general papers on DEM. In the literature, [6, 7] two approaches are described: the direct measurement approach, where parameters are measured directly at particle level. This can be difficult and correct results can only be expected if the DEM model uses an accurate representation of particle shape and size and if the contact law used takes into account all relevant physical effects. In contrast to this, the bulk calibration approach tries to bring measurements of the bulk material in accordance with simulation results of the DEM model by parameter variation. Such bulk calibration approaches can usually be found in the literature for railway ballast, e.g. [13, 15, 18, 37]. The bulk calibration approach has two potential problems. A minor problem is that it potentially weakens the physical meaning of the parameters. A major problem is that more than one combination of parameter values can result in the same (or similar) DEM simulation results. This problem is named parameter ambiguity and is addressed in detail in two current studies [2, 32]. Ref. [2] stresses the importance of checking for parameter ambiguity: without such checks it is questionable if a DEM model parametrised using one type of experiments, will be able to give reliable predictions to other experiments/applications. For the investigation of parameter ambiguity, a virtual calibration is conducted, where the DEM model is parametrised using simulation data instead of experimental data. In this way, the optimal parameter values are known beforehand and it is possible to check if these values can be found by the chosen parametrisation strategy or if parameter ambiguity exists. In [32], a different approach to reduce parameter ambiguity is suggested. For calibrating DEM models of cohesion-less free-flowing material, the coefficients of sliding friction and of rolling friction are sought. It is shown that the classical angle of repose test leads to very high parameter ambiguity: a large area in the 2D parameter space is identified, leading to the same angle of repose as seen in experiments. By using a so-called draw down test and by combining two angles and two mass flow rates (thus using more information from only one conducted experiment), the parameter ambiguity is dramatically reduced to a very small area in parameter space. The work done in [32] is continued in [31], where an optimisation based parametrisation procedure is presented.

**Fig. 1:**
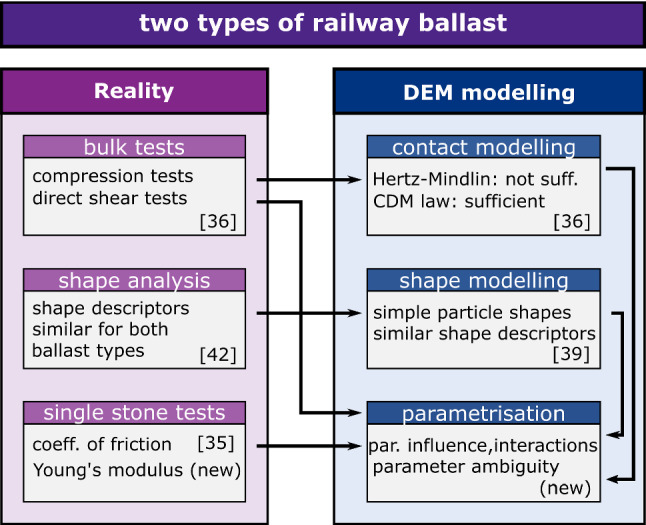
Sketch of previous and current work. Note that all measured data is openly available [35, 41, 43, 44]

*Related previous works* of the authors on two types of railway ballast, Calcite and Kieselkalk, are sketched as an overview in Fig. 1. Contact modelling was addressed in [37], comparing simulations with measured compression and direct shear tests. In the experiments, both types of ballast showed a very similar behaviour in the direct shear test, but clear differences could be seen in the uniaxial compression test. In [37], using a simple particle shape, clumps of three spheres, and applying the classical Hertz-Mindlin contact law, it was not possible to bring simulation results in good accordance with experimental measurements. However, using the CDM law, see [13], and applying a trial and error approach with quantitative error measures, for both types of ballast, one set of parameters could be found, such that simulations using simple particle shapes gave results in good agreement with the measurements.

A shape analysis with the same two types of railway ballast was the next step, [45], investigating 3D scanner data of ballast stones for several shape descriptors. No difference between the two types of ballast were found regarding flatness, elongation, roughness, sphericity, convexity index or a newly developed curvature-based angularity index. Thus, the observed difference in bulk material behaviour between Calcite and Kieselkalk are unlikely to be caused by differences in particle shape.

The knowledge on ballast shape was used in [42] to systematically construct simple particle shapes, which have similar shape descriptors as the real stones. Aiming for computational efficiency, clumps of three sphere were investigated and by analysing their packing behaviour, 20 simple particle shapes were found, which pack at the same porosities as Calcite and Kieselkalk ballast. In future, for these particle shapes a parametrisation to compression and direct shear tests needs to be conducted, which is a challenging task remembering that the CDM law has four parameters.

To facilitate this parametrisation process, in [36], cyclic measurements of the friction coefficient of the ballast stones of both Calcite and Kieselkalk were conducted. The results are summarised in the following Section and show a very high scatter.

*This work* is the next step to understand and speed-up the parametrisation process of simple particle shapes using the CDM law. Section 2 briefly summarises the experimental work carried out so far: compression and direct shear test and cyclic measurements of the coefficient of friction. Additionally, new measurements of the Young’s modulus of both Calcite and Kieselkalk are presented in this Section. Section 3 describes the details of the DEM simulation of compression and direct shear test. In Sect. 4, the influence of the four parameters of the CDM law on the simulation results are investigated. To do so, so-called characteristics of the simulation results are formulated, e.g. slope in the compression test or angle of dilation in the direct shear test. A detailed study of the influence of the parameters on these characteristics for both compression and direct shear test involves also simple statistical models for each characteristic. In Sect. 5, cost functions for the parametrisation are formulated based on the characteristics. A virtual calibration is conducted to investigate parameter ambiguity for each of the four parameters of the CDM law. With this knowledge the DEM model is parametrised to the experimental data. Suggestions for faster parametrisation (of similar particles shapes) are presented for a second particle shape. Conclusions are drawn in Sect. 6.

## Summary of experimental works

As in previous works, two different types of railway ballast will be considered: Calcite (stems from Croatia) and “Kieselkalk”, also known as Helvetic Siliceous Limestone, (stems from Switzerland). The types of ballast used are two out of five types tested at Graz University of Technology at the Institute of Railway Engineering and Transport Economy in the project “LoadLabs”,^1^ see [4] (in German, abstract available in English). In [3] (in German, abstract available in English), also mineralogical investigations were conducted. It was found that Calcite consists of 100% dolomite ((Ca, Mg) CO_3_), while Kieselkalk is composed of several constituents: 49,23% calcite (CaCO_3_); 37,63% quartz (SiO_2_); 5,54% dolomite ((Ca, Mg) CO_3_); 3,13% muskovite (KAl2(Si3Al)O10(OH,F)2); 1,79% albite (NaAlSi3O8); 0,85% pyrite (FeS2).

For these two types of ballast, uniaxial compression and direct shear tests, measurements of the coefficient of friction and of the Young’s modulus will be presented in the following Subsections.

### Compression and direct shear tests

Uniaxial compression tests as well as direct shear tests for both types of ballast were conducted and described in detail in [37], data is available at [41]. Here, a short summary of tests and their results will be given. Both compression and direct shear tests were conducted one after each other in a direct shear box test rig of the size 300 mm × 300 mm × 200 mm (shear box divided horizontally at medium height).

The densities (of the particles not of the bulk) of the material were measured, which gave 2822.2  kg/m^3^ for Calcite and 2660.0 kg/m^3^ for Kieselkalk. After filling in the ballast, it was precompacted using a vibrator compactor placed on a fitting wood board. Both types of ballast packed at similar porosities, between 0.425 and 0.46 (median value of 0.445).

**Fig. 2:**
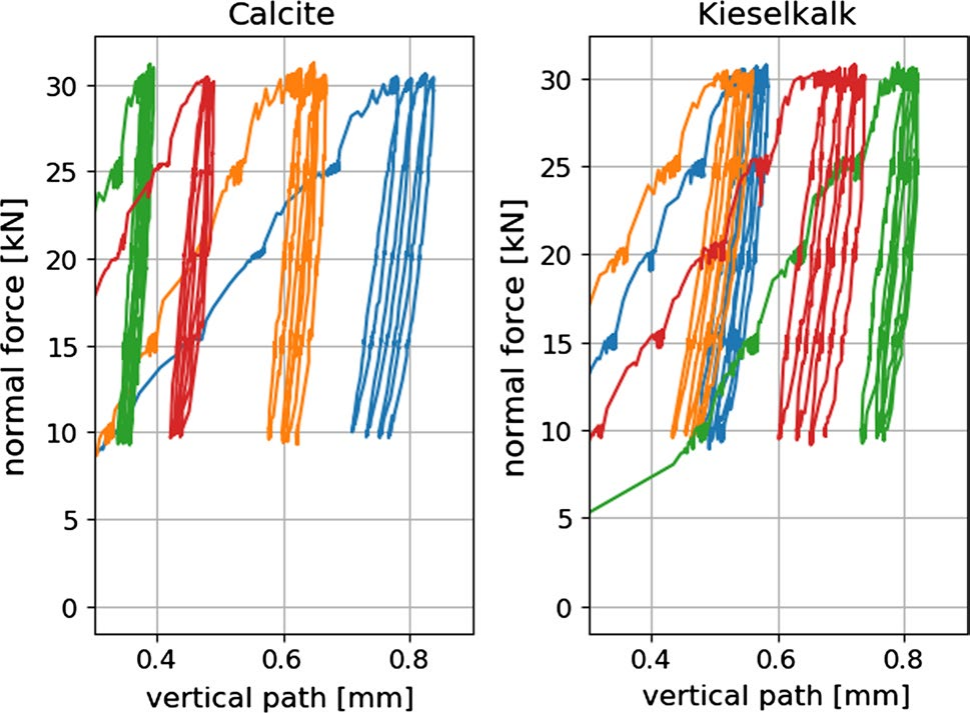
Examples of compression tests for the two types of ballast. Different colours correspond to repetitions of the same test

In the compression test, five different load levels were applied: 10, 15, 20, 25 and 30 kN. These values correspond to stresses between 111 and 333 kPa. Four load cycles were conducted and normal force as well as vertical displacement were measured. In total, nine compression tests were conducted for each type of ballast, Calcite and Kieselkalk. Typical results of measured normal force over vertical path can be seen in Fig. 2. Although all specimens were generated in the same way, the initial path until the first maximum in normal force is reached varies considerably for both types of ballast. This is not surprising as initially the load can be carried by only few ballast stones. Also the slope of the curves under loading shown in Fig. 2 varies, especially for Calcite.

**Fig. 3:**
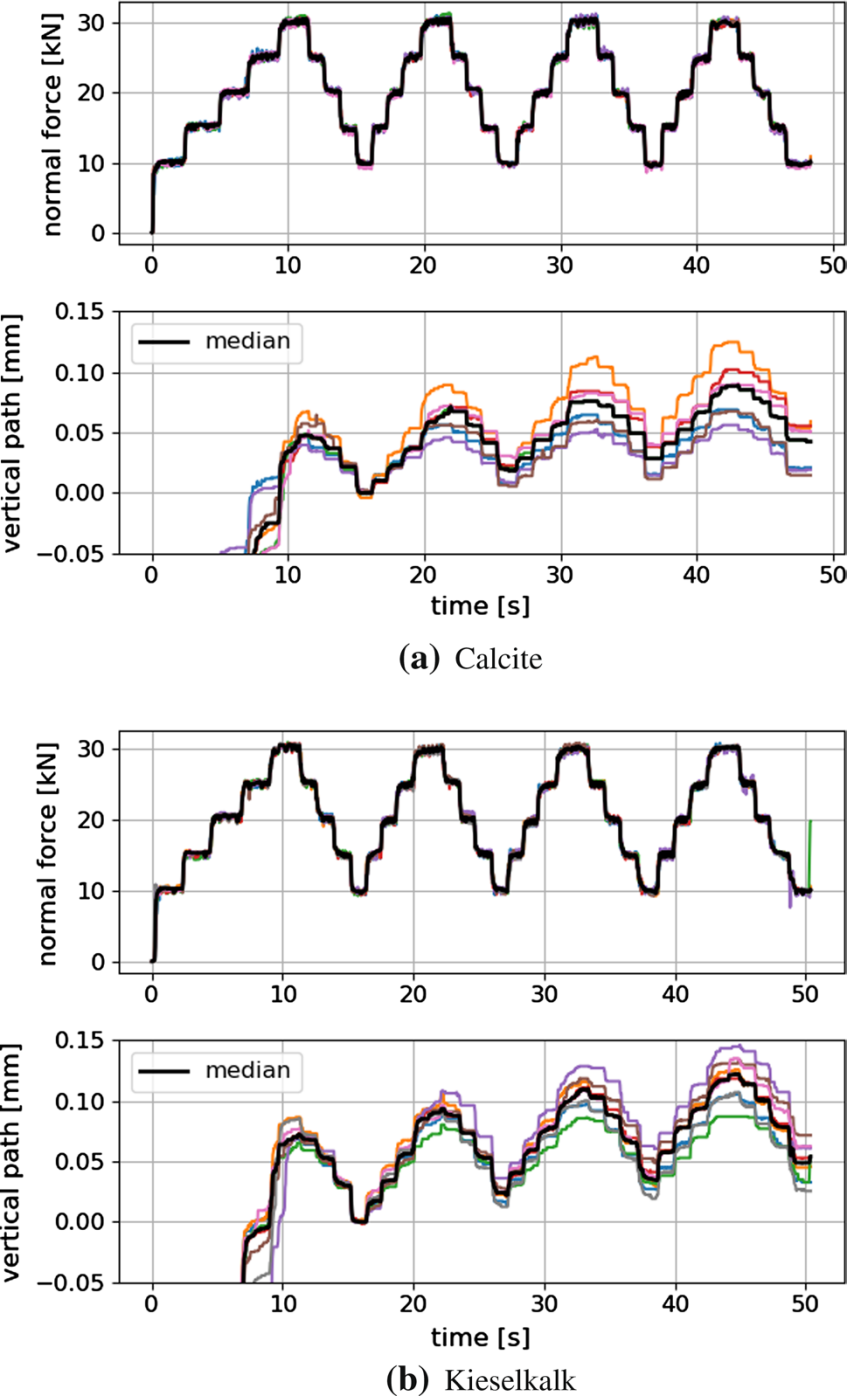
Time-warped path and force signals plotted over time, single measurements and median curve, for both types of ballast

**Fig. 4:**
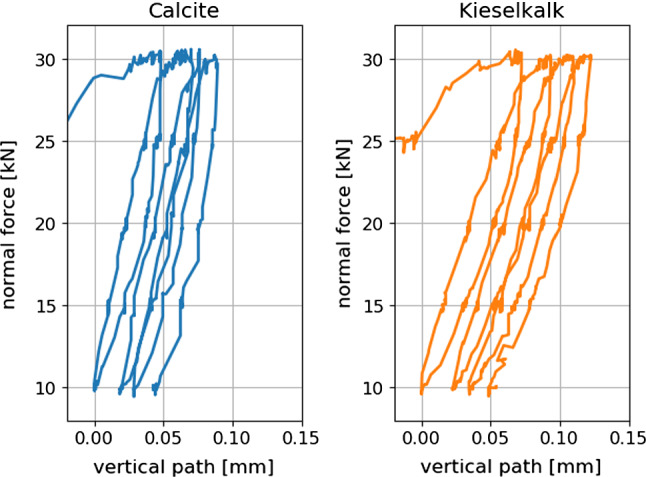
Median curves of normal force and vertical path of compression tests for both Calcite and Kieselkalk

Processing of this measured data is not straightforward. A so-called Dynamic Time Warping is applied to make measured force and path signals of different test comparable, see again [37] for details. Figure 3 shows the time-warped force and path signals for all measurements, also the median of the data is plotted. Due to the aforementioned strong scatter of the initial path, it was decided to shift the measured path to 0 after the first load cycle. In this representation, the scatter of the measured path can be seen also. The median curves of path and force are shown in Fig. 4 for both types of ballast. While they show similar settlement, i.e. final path, Calcite shows a considerably higher slope than Kieselkalk.

**Fig. 5:**
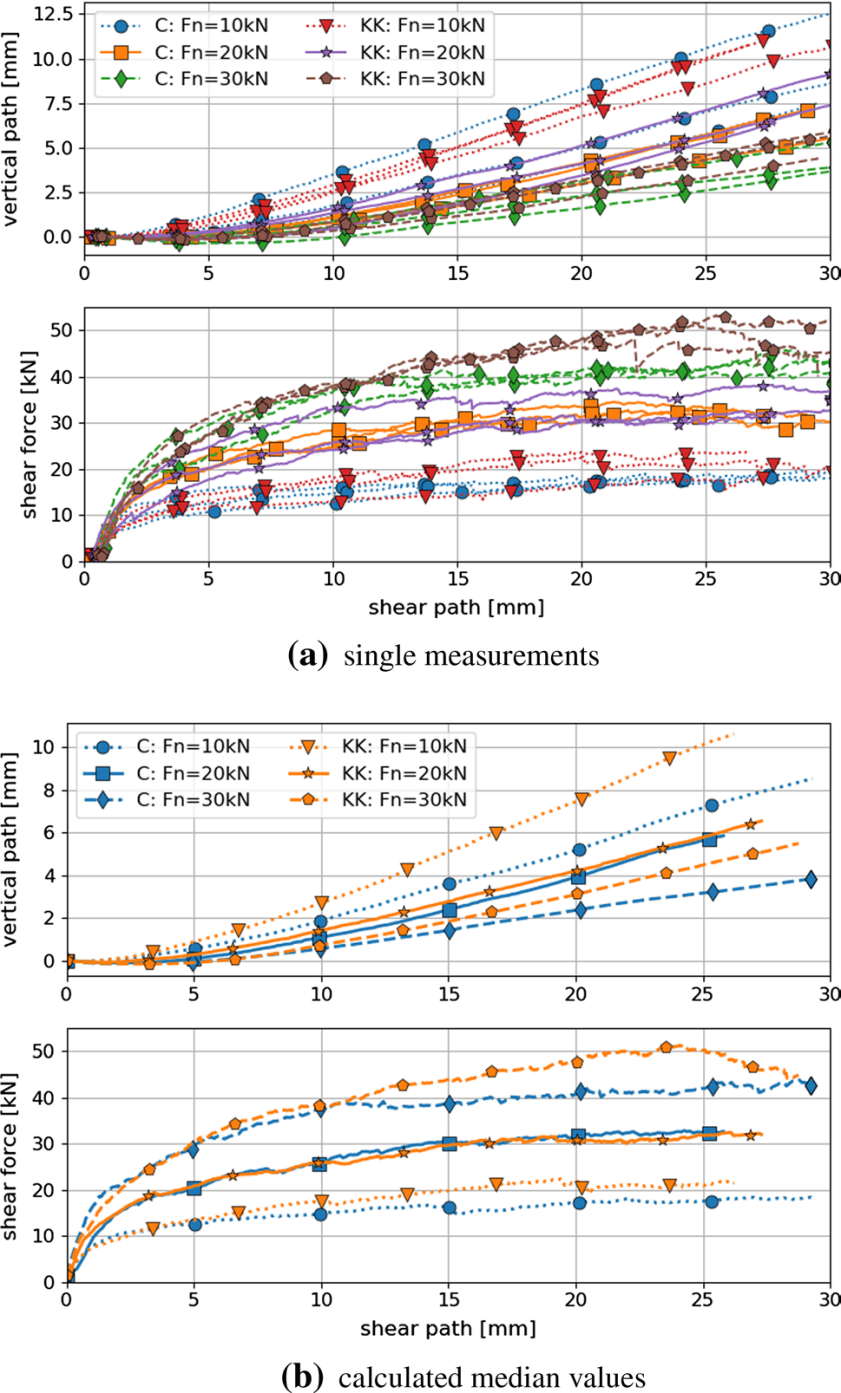
Shear force and vertical path plotted over shear path for Calcite (C) and Kieselkalk (KK)

The direct shear tests were conducted directly after the compression tests. Three different values of normal load were applied 10 kN, 20 kN and 30 kN and three tests were conducted for each level of applied normal load for both types of ballast. In Fig. 5a the shear force over the shear path and the compression dilation curves are shown for both Calcite and Kieselkalk. A direct comparison of both types of ballast can be seen in Fig. 5b, where the median is taken for each normal load separately. Despite the considerable differences in the behaviour of Calcite and Kieselkalk in the compression test, the results of the direct shear test are surprisingly similar. For the medium normal force, $F_n=20$ kN, the measurements are nearly identical for both types of ballast. However, for $F_n=10$ kN and 30 kN slight differences are observed, especially for shear paths larger than 15 mm. Although the specimens are rather dense with porosities around 0.445, the shear force curves do not show a clear maximum at the beginning of the test but grow monotonically. Regarding the measured normal displacement, all specimens showed a low initial contraction, followed by dilation. As expected, the lower the applied normal force the stronger is the dilation. Kieselkalk shows more dilation than Calcite, again the biggest difference is at the highest normal force.

### Measurement of coefficient of friction

Cyclic friction tests of both types of railway ballast were conducted and described in detail in [36], data is available at [35]. Here, a short summary will be given, focussing on the most relevant results. For the cyclic friction tests, an angular specimen (stone with a distinctive tip) was sheared over a flat specimen. The tests involved a loading phase up to a specified vertical load, shearing at constant velocity over a 10 mm shear distance and an unloaded return to the initial contact point. In this way, a given number of cycles were run for a specified vertical load. Then, the vertical load was increased and the testing continued until the cycling was finished at the highest vertical load. Three different test series were designed. In test series 1, five different vertical loads were investigated (10, 25, 50, 75, 100 N), while in test series 2, three different vertical loads were investigated (10, 50, 100 N). For both test series 1 and 2, 100 cycles per load were conducted. In contrast, in test series 3 only one cycle per vertical load was conducted for five different vertical loads (10, 25, 50, 75, 100 N).

**Fig. 6:**
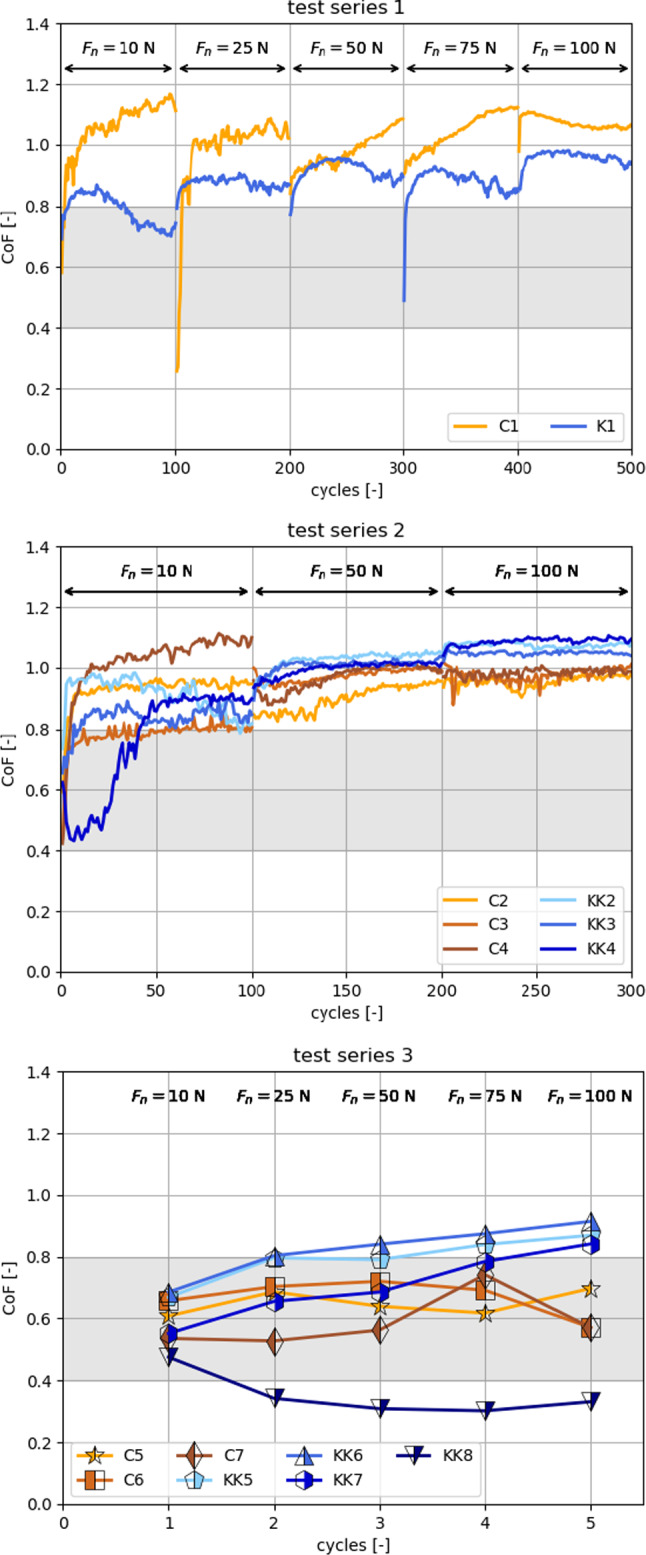
CoF plotted over cycles for both Calcite (C) and Kieselkalk (KK) for test series 1, 2 and 3. The grey shaded areas correspond to values of the CoF typically used in DEM simulations of railway ballast in the literature

The measured coefficient of friction (CoF) values of all test series are shown in Fig. 6. Three main observations can be made. First, when comparing Calcite and Kieselkalk no clear difference can be seen between the measured CoF values. In test series 1, CoF values measured for Calcite are mostly higher than those for Kieselkalk, while the opposite trend can be seen in test series 2 and 3. (With the exception of test KK8, which consists of the lowest CoF values measured. No obvious explanation for the different behaviour of this test exists.) The second observation from Fig. 6, is the high amount of scatter in the measurements. As stones are a natural material, the non-homogeneous composition or the stone’s geometry might be a reason, but also the test rig’s control system may have an effect. The third observation is that in test series 1 and 2, which included 100 cycles for each level of vertical load, higher CoF values were measured than in test series 3, where only one cycle per vertical load was conducted. A detailed discussion of possible tribological mechanisms can be found in [36].

When looking in the literature, CoF values in the range of 0.4–0.8 are typically used for DEM simulations of railway ballast, [13–25, 29, 37, 47]. In the experiments presented above, this range is mainly covered by the results for tests series 3 with only one cycle—see the grey shaded areas in Fig. 6. In test series 1 and 2, the measured CoF values were (almost all) higher than 0.8 after a few cycles. When looking at ballast in real track, millions of load cycles occur. Here, higher CoF values may exist according to these findings.

The relatively low values used in DEM simulations—0.4 to 0.8 comparable to test series 3—are not surprising. For parametrisation and validation of DEM models, direct shear tests or monotonic triaxial tests are frequently used, see e.g. [16, 23, 25, 29, 37]. In these tests, always fresh stones come into contact, which might be a comparable situation to test series 3. The calibrated DEM models are then used to simulate cyclic loading, e.g. in box tests [5, 21], to investigate load cases closer to track conditions. In the cyclic loading in box tests or at track sites, contact partners stay mostly the same and are sheared repeatedly over each other. This scenario is quite similar to the cyclic friction tests of series 1 and 2, where CoF values between 0.8 and 1.2 were measured. The measurements conducted indicate that DEM models calibrated using direct shear tests or monotonic triaxial tests might have used CoF values that were too low, when cyclic loading is applied, e.g. in a box test.

Thus, more sophisticated friction models for DEM simulations might become necessary, if both cyclic and non-cyclic shearing is to be simulated.

### Measurements of Young’s modulus

**Fig. 7:**
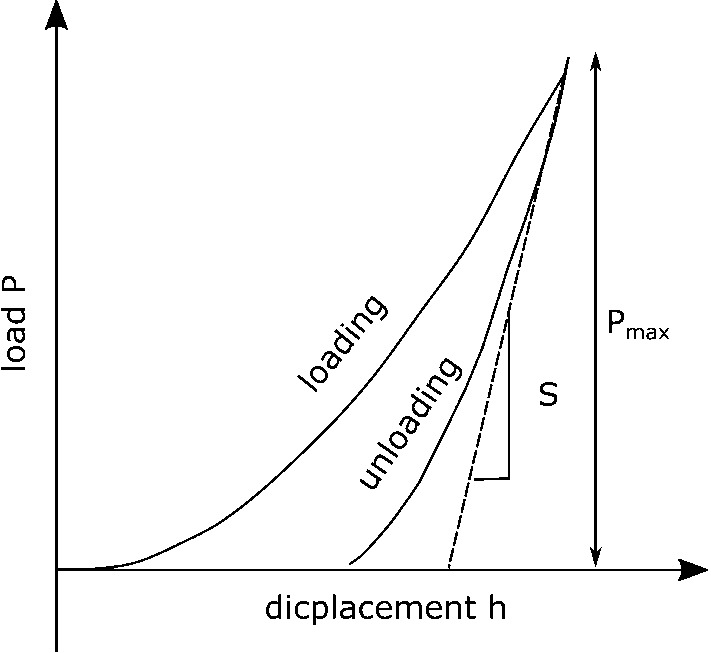
Sketch of load displacement curve for an indentation experiment, redrawn from [26]

For each type of ballast, the Young’s modulus was measured on three separate stones via nano-indentation. The measurement data is openly available [44]. The machine used was a Hysitron TI Premier. Rock samples were set in epoxy resin and subsequently ground and polished to achieve a level surface with a maximum roughness of  1 $\mu$m before 40 indentations were made into each sample with a Berkovich (pyramidal) indenter tip. For the indentations, the following load function was used: 5 s of ramping up to 10,000 $\mu \text{ N }$ (1000 $\mu \text{ N }$ for the first pair of rock samples, “K1” and “KK1” (Young’s modulus measurements are independent of load)), 5 s dwell time, then 5 s force unloading. The outputted load displacement curve, see Fig. 7 for a sketch, was then analysed according to the Oliver-Pharr Method, [26].

In a first step, the reduced modulus, $E_r$ is calculated via:

1 $$\begin{aligned} S=\frac{dP}{dh}=\frac{2}{\sqrt{\pi }}\, E_r\, \sqrt{A}, \end{aligned}$$

where $S=\frac{dP}{dh}$ is the experimentally measured stiffness of the upper portion of the unloading curve and *A* is the projected area of the contact (a function of contact depth for a pyramidal tip). Then, the Young’s modulus, *E*, is calculated, making an assumption of the material’s Poisson’s ratio, here $\nu =0.3$ is assumed,

2 $$\begin{aligned} \frac{1}{E_r}=\frac{(1-\nu ^2)}{E} + \frac{(1-\nu _i^2)}{E_i}, \end{aligned}$$

where $E_i$ and $\nu _i$ are the Young’s modulus and the Poisson’s ratio of the indenter.

**Fig. 8:**
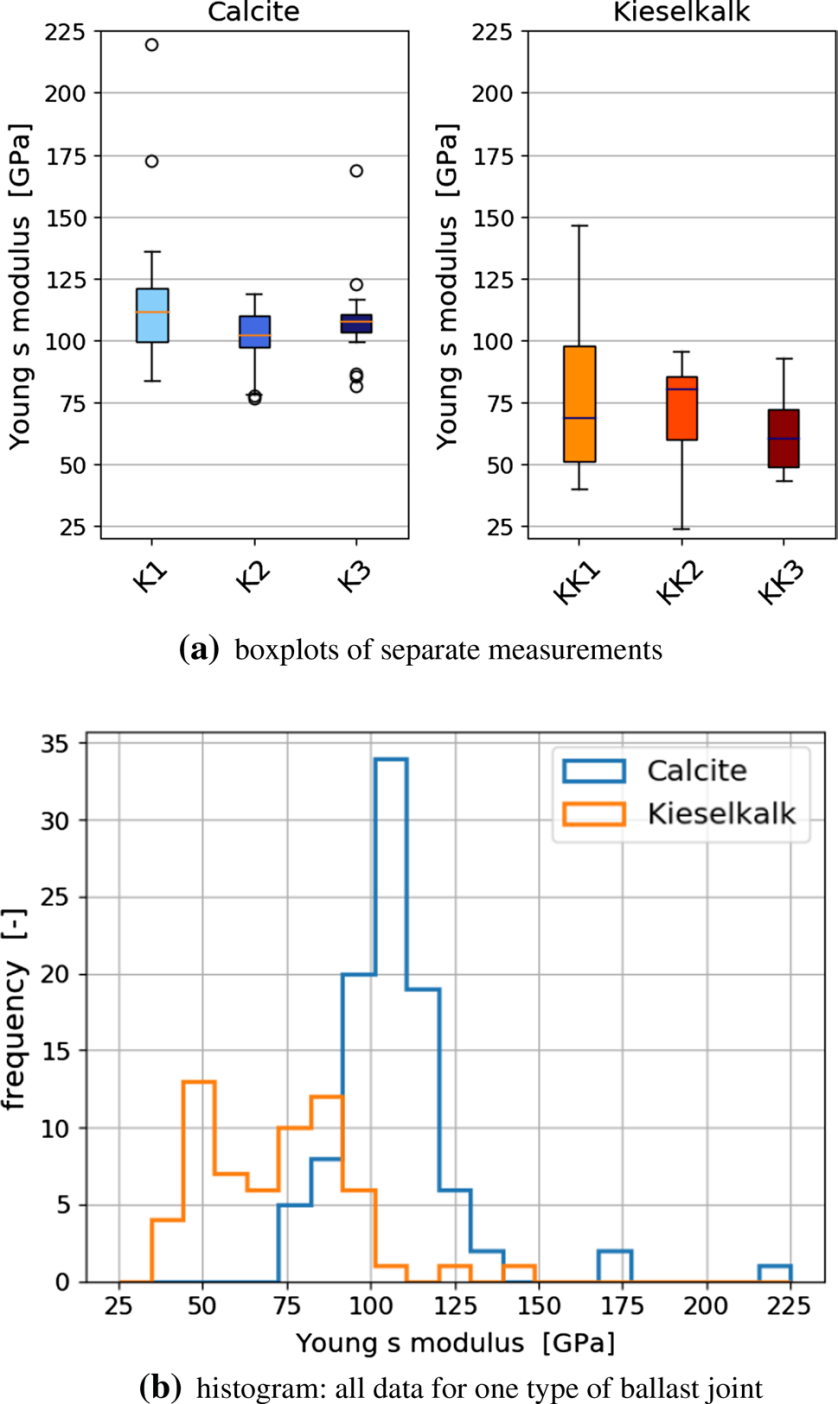
Measured values of Young’s modulus for Calcite and Kieselkalk

The calculated values of the Young’s modulus are shown in Fig. 8a as box plots for the individual rock samples of both types of ballast. It can be seen that for Calcite notably higher Young’s moduli values were calculated, compared to Kieselkalk. Also, for Calcite the obtained values are concentrated in a narrower range, while the results for Kieselkalk spread over a broader range. This can be seen even better in Fig. 8b, where all measured values for each type of material are plotted as a histogram. The scatter in results can be expected as these are local measurements that are taken to give a global understanding of the Young’s modulus. Some indentation points had to be deleted based on the outputted data. Here, problems occurred that may arise when indenting into local porosities, dirt or grain boundaries. As mentioned at the beginning of the Section, Kieselkalk is made up of different materials, so results can change based on the constituent material being indented into. In contrast, Calcite consists purely of dolomite, which explains the smaller range of obtained results.

## DEM simulation details

For all DEM simulations in this work the software YADE [48] will be used. It is Open-Source and utilises the soft contact approach together with explicit integration in time. As mentioned in the introduction, previous work of the authors included particle shape analysis on the two types of ballast, [45]. Aiming for computational efficiency, a number of simple particle shapes (clumps of three spheres) were constructed, which have similar shape descriptors as the real ballast stones, see [42]. Investigating packing behaviour, 20 particle shapes were identified, which could pack at similar porosities as seen in the experiments. For simplicity, these samples were generated using the classical Hertz-Mindlin contact law because only their packing behaviour was studied.

### Used contact model: the CDM law

In this work, the CDM contact law will be used based on the experiences gained in [40] and [37] with the simulation of the compression and direct shear test. The CDM law, developed in [13], is an extension of the Hertz-Mindlin model, where a kind of ideal plasticity is is introduced to model damage at a contact (e.g. to take into account edge breakage), which turned out to be necessary to describe the observed behaviour in the experiments.

When two spheres (or analogously a sphere and a wall) come into contact, the geometric overlap calculated by the DEM software, $\delta _{{\textsc {DEM}}}$ is split in an elastic part, $\delta _{el}$, and a plastic part, $\delta _{pl}$: $\delta _{{\textsc {DEM}}}= \delta _{el} + \delta _{pl}$. Here, $\delta _{pl}$ is initialised with zero when the contact is created. In the elastic regime, the normal force is calculated with the Hertz law

3 $$\begin{aligned} F_n = \frac{4}{3} E^* \sqrt{R} \left( \delta _{el}\right) ^\frac{3}{2} \; , \end{aligned}$$

where $E^*$ is the equivalent Young’s modulus and *R* is the current radius in the contact which is initialized with the equivalent radius $R^*$. The maximal stress at the contact, $\sigma _0$, can be calculated as

4 $$\begin{aligned} \sigma _0 =\frac{2 E^*}{ \pi } \sqrt{ \frac{\delta _{el}}{R} } \; . \end{aligned}$$

This stress is limited with a pseudo maximal compressive strength, $\sigma _{{\mathrm{max}}}$. If $\sigma _0 > \sigma _{{\mathrm{max}}}$, the stress is too high for the material to be carried and damage/plastic yielding occurs. The spheres in contact are thought to flatten locally, thus *R* and $\delta _{pl}$ increase such that $\sigma _0=\sigma _{{\mathrm{max}}}$. The relation between $\delta _{pl}$ and *R* are derived in [13] such that

5 $$\begin{aligned} \delta _{pl} =(R - R^*) \beta , \end{aligned}$$

where the material parameter $\beta$ relates to an opening angle of a conical asperity $\alpha$ as: $\beta = \frac{1-\sin (\alpha )}{ \sin (\alpha )}$. From the limitation of the stress $\sigma _0=\sigma _{{\mathrm{max}}}$ in the plastic case, it follows:

6 $$\begin{aligned} R=\frac{ \delta _{{\textsc {DEM}}}+ R^* \beta }{ \left( \frac{ \sigma _{{\mathrm{max}}}\pi }{2 E^*}\right) ^2 + \beta } . \end{aligned}$$

**Fig. 9:**
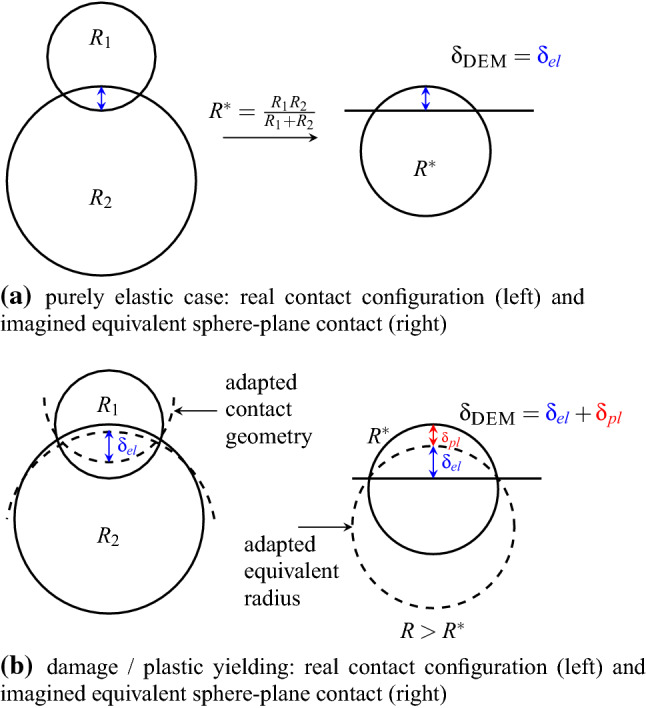
Conical damage model for a sphere-sphere contact

With this equation it is possible to solve the model accurately without the need of an iterative procedure, see [40] for details. Figure 9 shows a schematic drawing of the initially elastic contact, Fig. 9a, and the plastic yielding, Fig. 9b. Note that the actual radii of the spheres in the DEM model remains unchanged, just the radius of the contact area, *R*, increases in the contact law. In this formulation of the CDM law, the calculation of the tangential force remains unchanged from the Hertz-Mindlin law. In total the CDM law has four parameters: *E*, $\sigma _{{\mathrm{max}}}$, $\alpha$ and $\mu$.

### Sample generation and pre-compaction

As a first step, the initial samples, generated with Hertz-Mindlin law from [42], were used and the contact law was changed to the CDM law. In this way, it was ensured that the simulations using different parameter sets had the same initial porosity. Problems occurred in the simulations of the compression test, where an unrealistically high vertical displacement was obtained until the maximal force of the first load cycle was reached. Consequently, the specimen’s porosity at the end of the compression test (which is the start of the direct shear test) was much too low compared to the experiments.

To solve this problem, samples were generated using the CDM law in a two step procedure. First, a given set of parameters was used and particles were filled in the shear box in a rainfall procedure. After settling, all particles above the box were deleted. The initial friction coefficient, $\mu _{{\mathrm{ini}}}$, was varied, such that the mass of the sample in the box is similar to the mass of the experimental specimens ($\approx 26$ kg for Kieselkalk ballast). This initial configuration was saved to a file before pre-compaction.

For the second step of pre-compaction, this file was loaded and the contact parameters were set to their final values, except $\mu =\mu _{{\mathrm{ini}}}$. A normal load was applied on the sample until a porosity of 0.445 was reached (median values of experimental specimens), then the sample was unloaded. The normal load necessary to reach the target porosity was strongly dependent on the used parameters. However, this load was in all cases lower than the maximal load applied in the compression test. After unloading, the coefficient of friction $\mu$ was set to its final value.

Analysing the internal properties of the packing it showed that this procedure of specimen generation caused a considerable yielding of contacts. This lead to an increase of the contacts’ radii *R*, which in turn caused a much stiffer response, i.e. reduced vertical displacement, in the compression test. This procedure of sample generation and pre-compaction ensures that simulations using different sets of parameters all have the same mass and a similar initial porosity.

**Fig. 10:**
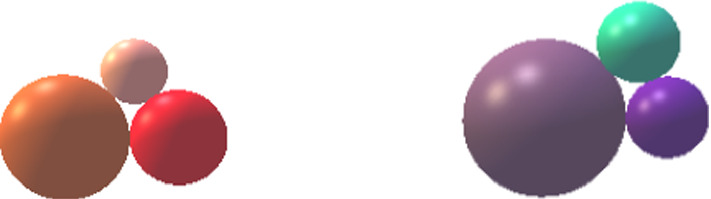
Used clump shapes in this paper. Left: shape no. 7. Right: shape no. 9 (used later on)

In this work, at first the following particle shape will be considered: shape no. 7 from [42], which is a clump of three non-overlapping spheres of the radii 8.53, 6.47 and 4.41mm. The generated samples consist of roughly 2500 particles. Fig. 10 shows an image of clump shape no. 7 and shape no. 9 (used later on). An investigation of the influence of the parameters of the CDM law *E*, $\sigma _{{\mathrm{max}}}$, $\alpha$ and $\mu$ on the simulation results of Kieselkalk ballast will be shown in the next Section. All remaining material parameters, e.g. for the Hertz-Mindlin law used for the steel box, are given in Table 1.

**Table 1:** Material parameters used for the simulations

	*E* (GPa)	$\nu (-)$	$\mu (-)$	$\sigma _{{\mathrm{max}}}$ (MPa)	$\alpha (^\circ)$	$\rho$ (kg/m$^3$)
Kieselkalk	id.	0.2	id.	id.	id.	2660.0
Steel box	200	0.28	0.2	–	–	7833.34

Parameters, which are to be identified, are denoted with “id.”

## Influence of parameters on simulation results

A good understanding of the model parameters’ effect on the simulation results is considered of high importance. Therefore, it was chosen to conduct simulations with parameters belonging to a full Design of Experiments (DoE) and to analyse the results in detail. With the number of levels considered for each parameter, the necessary number of simulation runs can be controlled. An advantage of a full DoE plan is that interactions between parameters can be studied. If the number of conducted simulation runs needs to be small, also other sampling methods can be applied, e.g. factorial designs, Placket-Burman designs or Latin Hypercube sampling, compare e.g. [31] for different sampling approaches.

The levels of the full DoE applied here are given in Table 2. The results of the measured Young’s modulus, see Sect. 2.3, cover a range between 25 and 150 GPa, which is plausible considering that Kieselkalk ballast consist of many constituents. Here, it was chosen to limit the investigation to the central 50% of the measured values of the Young’s modulus, which gives a range for *E* from 50 to 85 GPa. The parameter $\sigma _{{\mathrm{max}}}$ is the pseudo maximal compression strength, and thus cannot be measured. It describes how much stress the material can carry and it therefore always acts in combination with the Young’s modulus. As in the calculation of the CDM model the ratio of $E/\sigma _{{\mathrm{max}}}$ is used, this ratio will be included in the DoE. Parameter $\alpha$ describes the angle of asperities in the CDM model. It influences the (virtual) growth of the contact’s radius during yielding, however a direct link to measurements is difficult. The considered range for $\alpha$ is derived from previous experience with the CDM model. For the coefficient of friction, $\mu$, the measurements summarised in Sect. 2.2 showed a high amount of scatter. Therefore, it was decided to choose values for $\mu$ in a similar range, as in previous work, where similar particle shapes were considered. These values of $\mu$ lie in the range of the measured CoF values of test series 3 (only one cycle per load level). The chosen DoE design results in a total of 150 simulation runs, which can be conducted in parallel. Three different initial positions of the particles were investigated resulting in scatter when comparing the results.

**Table 2:** Parameters and their used levels in the full DoE plan

Parameter					
*E* (GPa)	50	67	85		
$E/\sigma _{{\mathrm{max}}}(-)$	50	100	150	200	250
$\alpha$ ($^\circ$)	81	83	85	87	89
$\mu (-)$	0.4	0.5			

**Fig. 11:**
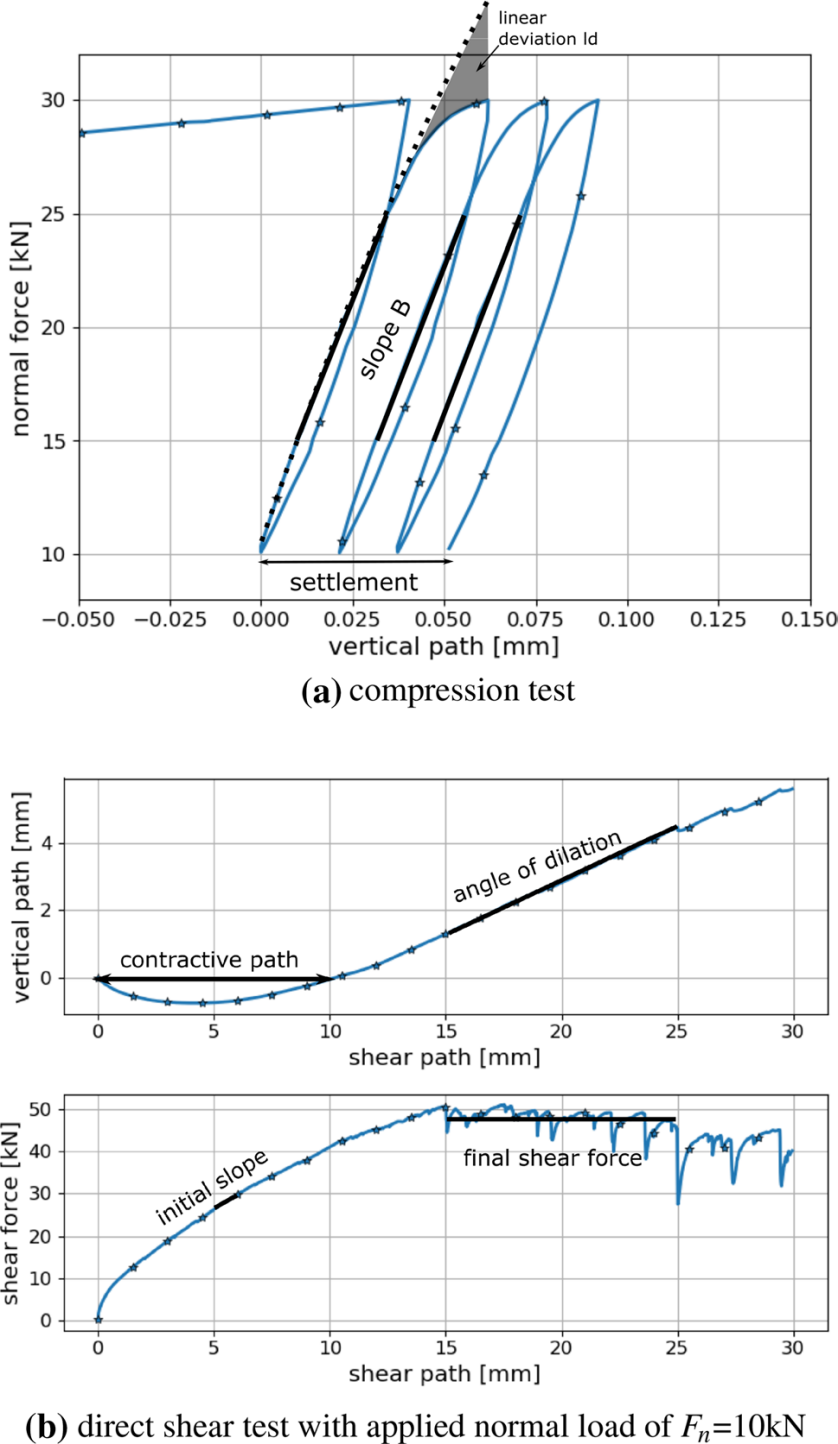
Simulation results for compression and direct shear test, including sketches of characteristics

### Characterising simulation results

To quantify the influence of different parameter sets on the simulation results, so-called characteristics will be formulated. Example simulation results for one set of parameters are shown in Fig. 11, for both compression and direct shear test. In the Figure the characteristics are also depicted, which will be introduced in detail in the following. For the compression test, the first obvious characteristic is the slope of the curve under loading. This slope is denoted as *B* and is calculated by fitting lines between 15 and 25 kN for load cycles two, three and four and by taking the median of the slopes of these lines. Furthermore, the shapes of the curve under loading for load cycle two will be considered. The deviation from the linear fit, denoted as $l_d$, will be calculated as follows, compare also Fig. 11a,

7 $$\begin{aligned} l_d=\sqrt{\frac{1}{x_2-x_1} \int _{x_1}^{x_2} \left( l(x) -s(x) \right) ^2 dx }, \end{aligned}$$

where $x_1, x_2$ denote the vertical path belonging to the minimum and maximum force, *l*(*x*) the fitted line in the force-path diagram and *s*(*x*) the simulated force-path curve. Finally, using the vertical path between cycle two and cycle four, the settlement will be considered.

To characterise the results of the direct shear test simulation, the angle of dilation, $\psi$, will be calculated by fitting a line to simulated vertical path between 15 and 25 mm shear path. At the beginning of the shear test, the sample usually undergoes contraction, i.e. the measured vertical path is negative. The length of this path is called contractive path. The final shear force, $s_f$, is calculated as the median of the simulated shear force between 15 and 25 mm shear path. The initial slope of the shear path-shear force curve is calculated by fitting a line between 0.5 and 1 mm of the shear path.

### Characteristics of compression test

**Fig. 12:**
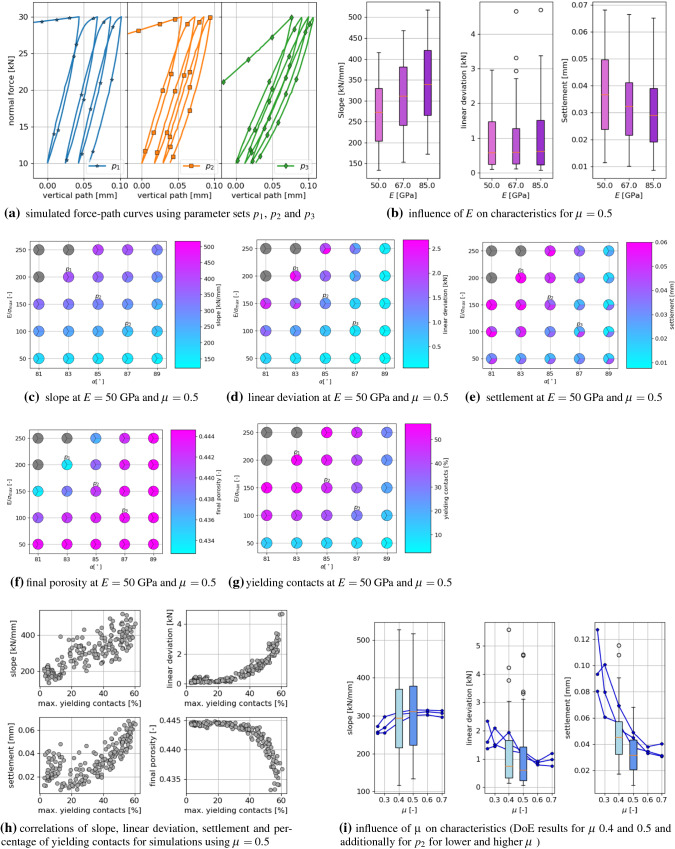
Influence of parameters *E*, $E/\sigma _{{\mathrm{max}}}$, $\alpha$ and $\mu$ on simulated compression test: characteristics

The simulation results of the compression tests are evaluated for the characteristics: slope, linear deviation and settlement. The influence of the parameters *E*, $E/\sigma _{{\mathrm{max}}}$, $\alpha$ and $\mu$ on these characteristics can be seen in Fig. 12. To visualise the considered characteristics, three parameters sets, $p_1, p_2$ and $p_3$ (see e.g. Fig. 12c for the corresponding parameter values), were chosen and the resulting force-path curves are plotted in Fig. 12a. Parameter set $p_1$ leads to the highest slope, linear deviation and settlement. For the remaining parameter sets $p_2$ and $p_3$, the slope reduces, as well as the linear deviation, i.e. the curves look more linear under loading, and also the settlement reduces. To investigate the influence of *E*, Fig. 12b shows box plots of the three characteristics for the three different values of *E* using $\mu =0.5$. The calculated slope values show a moderate increase with increasing *E*, which would also be the case for the purely elastic Hertzian model. In contrast, the calculated values of the linear deviation show nearly no dependence on *E*. The settlement values show a slight decrease with increasing *E* values.

For a more detailed investigation, Fig. 12c–e show the influence of the parameters $E/\sigma _{{\mathrm{max}}}$ and $\alpha$, while $E=50$ GPa and $\mu =0.5$ are fixed (the corresponding plots for different values of *E* or $\mu$ look qualitatively similar). These plots show for each parameter set the results of three simulations with different initial configurations as a pie chart, to give a good visual understanding of the amount of scatter. In each of these Figures, three points are coloured in grey: the following parameter sets $E/\sigma _{{\mathrm{max}}}=250$, $\alpha =81^\circ$ and $E/\sigma _{{\mathrm{max}}}=250$, $\alpha =83^\circ$ and $E/\sigma _{{\mathrm{max}}}=200$, $\alpha =81^\circ$ lead to an unrealistic high initial path in the compression test, therefore these results are skipped. The reason for this behaviour can be explained with the details of the CDM law, see [40] for all equations and the computational algorithm.

From the three considered characteristics in Fig. 12c–e, the slope shows little scatter stemming from different initial configuration (similar colours in each circle/each parameter set), while more scatter can be seen for the linear deviation and the strongest scatter is seen for the settlement. For all three characteristics, the influence of $E/\sigma _{{\mathrm{max}}}$ and $\alpha$ is qualitatively similar, with high values for high $E/\sigma _{{\mathrm{max}}}$ and low $\alpha$ and dropping values for decreasing $E/\sigma _{{\mathrm{max}}}$ and/or increasing $\alpha$. Figure 12f, g shows the same type of plot for the final porosity and the percentage of yielding contacts at the maximal load of cycle 2. The final porosity will be of interest later, as it determines the initial porosity for the direct shear test. All samples are generated with a porosity of about 0.445 and it can be seen that the porosity decreases only slightly during the test for parameter combinations including low values of $E/\sigma _{{\mathrm{max}}}$ or high $\alpha$. These are also the parameter sets with a low percentage of yielding contacts. On the contrary, the lowest final porosities can be seen for parameter combinations of high $E/\sigma _{{\mathrm{max}}}$ and low $\alpha$ values, which correspond to a high percentage of yielding contacts.

To further investigate the influence of the yielding contacts, Fig. 12h shows correlation plots. Plotted are the three characteristics, slope, settlement, linear deviation and the final porosity over the percentage of yielding contacts at the maximal load of cycle 2. The linear deviation and the final porosity shows a very strong and non-linear correlation to the percentage of yielding contacts. For the settlement this correlation is weaker (also non-linear) and for the slope only a weak and linear correlation to the yielding contacts can be seen.

A material behaviour close to the elastic one is seen for parameter sets, which cause only a small percentage of yielding contacts. These result in low slopes and nearly linear shape under loading, i.e. low linear deviation, and (due to high scatter) settlement up to 0.04 mm. Moreover, they result in small changes in sample porosities, caused mainly be particle rearrangement and energy is dissipated mostly through sliding. These mentioned parameter combinations include low values of $E/\sigma _{{\mathrm{max}}}$ or high $\alpha$. Plasticity is involved in the material’s behaviour for parameter sets, which cause many yielding contacts. Here, simulation results with higher slopes, high linear deviation and also higher settlement are seen. These parameter sets result in samples with the lowest final porosities, as in yielding contacts the plastic overlap $\delta _{pl}$ increases, which does not cause repulsive forces. The plastic yielding can be expected to be the main mechanism of energy dissipation. Parameter combinations with many yielding contacts involve high $E/\sigma _{{\mathrm{max}}}$ and low $\alpha$ values.

Finally, the influence of $\mu$ on the characteristics is shown in Fig. 12i. Box plots are shown for simulation results obtained from the DoE with $\mu$ equals 0.4 and 0.5 (150 parameter sets). Additionally, line plots show results, where $\mu$ was varied on a broader range, from 0.25 to 0.7 for one single parameter set $p_2$ for three different initial configurations. Increasing the value of $\mu$ from 0.4 to 0.5, leads to a slight increase in slope as well as a slight decrease in the linear deviation. A bigger influence of $\mu$ is seen for the settlement, where lower values are seen to be caused by higher values of $\mu$, as one would expect. The line plots cover the range of 0.25–0.7 for $\mu$, thus, its influence on slope, linear deviation and the settlement can be seen more clearly. For the lowest values of $\mu =0.25, 0.3$ also the highest scatter in the results is seen, as it can be expected.

**Table 3:** Compression tests: statistically significant predictors and $R^{2}$ value of simplified linear models for the characteristics: slope, linear deviation and settlement

	Slope	Lin. dev.	Settlement
*E*	x		x
$E/\sigma _{{\mathrm{max}}}$	x	x	x
$\alpha$	x		
$\mu$	x	x	x
I($E^2$)			
I($(E/\sigma _{{\mathrm{max}}})^2$)	x	x	x
I($\alpha ^2$)	x	x	x
I($\mu ^2$)			
I($E \cdot E/\sigma _{{\mathrm{max}}}$)	x		
I($E \cdot \alpha$)	x	x	
I($E \cdot \mu$)		x	x
I($(E/\sigma _{{\mathrm{max}}}) \cdot \alpha$)	x	x	x
I($(E/\sigma _{{\mathrm{max}}}) \cdot \mu$)			x
I($\alpha \cdot \mu$)	x		x
$R^{2}$	0.97	0.86	0.56

As an additional investigation, a statistical analysis is conducted. The calculated characteristics and their corresponding parameter sets are loaded into the open-source statistical software R, [30]. As a first step, a linear model is fitted through the data using R’s +lm+ function for each characteristic. The model is always the same and includes the following predictors: the four parameters, $E, E/\sigma _{{\mathrm{max}}}, \alpha , \mu$, the squared influence of these four parameters, written in R notation as, I($E^2$), I($(E/\sigma _{{\mathrm{max}}})^2$), I($\alpha ^2$), I($\mu ^2$) and all first order interactions , I($E \cdot E/\sigma _{{\mathrm{max}}}$), I($E \cdot \alpha$), I($E \cdot \mu$), I($(E/\sigma _{{\mathrm{max}}}) \cdot \alpha$), I($(E/\sigma _{{\mathrm{max}}}) \cdot \mu$), I($\alpha \cdot \mu$). In a backwards stepwise regression, R’s +step+ function removes the least contributive predictors, and stops when all remaining predictors are statistically significant. The resulting simplified models for each characteristic can be seen in Table 3, where the significant predictors are marked with an “x”. Here, also the $R^{2}$ values of the fitted models are given. The linear model fitted to the slope values contains as predictors all four parameters, as well as the squared influences of $E/\sigma _{{\mathrm{max}}}$ and $\alpha$ and four out of the six interactions between the parameters. With an $R^{2}$ value of 0.97 this model fits the data very well. Only very little scatter of the data exists, which cannot be explained by this model. The model fitted to the linear deviation consists of less predictors, but also fits the data less well. From the four parameters, in the predictors the following are included: $E/\sigma _{{\mathrm{max}}}$ (simple and squared), $\alpha$ (squared) and $\mu$ (simple). Confirming the conclusion drawn from Fig. 12b, *E* is not a predictor of this model. After all, the influence of *E* is included in two interactions. The $R^{2}$ value of this model is with 0.86 lower than the one for the slope but still gives an acceptable quality of the fit. Finally, the model for the settlement contains all four parameters (either simple or squared contribution) as predictors. Also from the interactions, four out of six are contained in the model. Although the number of predictors is quite high, the $R^{2}$ value of 0.56 indicates a poor fit for the model. This is attributed mainly to the observed scatter of the settlement data, although a general misfit between model and data (e.g. missing predictor or different form of model) cannot be ruled out completely. Summing up, it is shown that the three characteristics are influenced directly by all four parameters (with the exception that the linear deviation is not influenced by *E*). The interaction between $E/\sigma _{{\mathrm{max}}}$ and $\alpha$ is also described in the Fig. 12c till 12e, but several other interactions could be identified from this analysis. The interplay of parameters and their interactions is thus highly complex.

### Characteristics of direct shear test

**Fig. 13:**
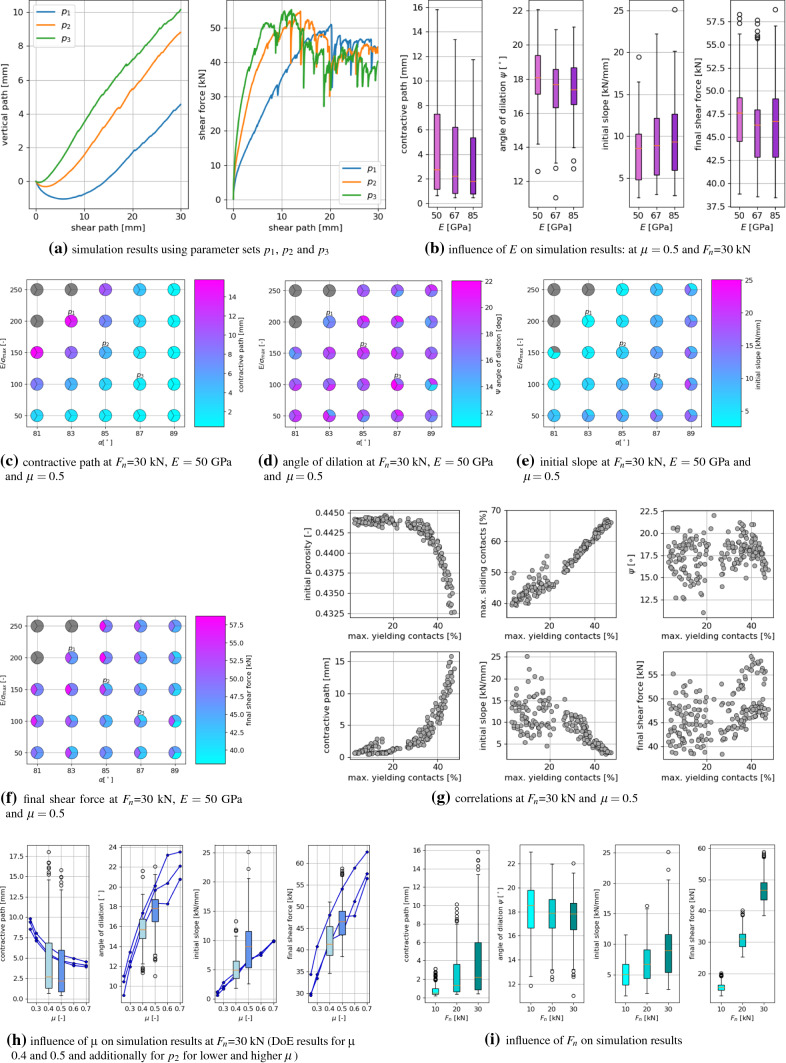
Influence of parameters on simulated direct shear test: characteristics

After the compression test, the simulation results of the direct shear test are analysed in a similar manner in Fig. 13. To give a good visual impression, Fig. 13a shows the simulated dilation curve, i.e. vertical path, and the simulated shear force for the three parameter sets $p_1, p_2$ and $p_3$ (the same as mentioned before for the compression test). For these three parameter sets, the contractive path differs strongly, while the dilation angle $\psi$ is similar. The initial slope of the shear force also shows big differences for the three different parameter sets. In the further course of shearing, the shear force-path curve includes sudden jumps, where the shear force builds up and then suddenly drops. It is assumed that this behaviour is caused by the rigidity of the DEM particles. While real ballast stones can break, the DEM particles are rigid during shearing in this test. Under shearing, the particles shape gives raise to interlocking, also called “geometric friction” see [19, 33]. When interlocking occurs, the shear force is expected to increase until the particles suddenly start sliding and the shear force suddenly drops. This process obviously depends on the coefficient of friction $\mu$. The rigidity of DEM particles is also discussed in [28]. Here, monotonic triaxial tests of railway ballast were simulated using polyhedral elements. Using different initial configurations in the DEM simulations showed big differences in the resulting stress-strain curves. This was explained in [28] with: “The rigid particles used do not break or chip, which can cause the simulated stress-strain response to include sudden jumps, referred to as peaking up or dropping”. This is a possible explanation for rather big differences in the simulated shear force belonging to different initial configurations, seen in the following.

Figure 13b shows box plots of the four characteristics of the shear test depending on the Young’s modulus *E* for the highest applied normal load $F_n=30$ kN and $\mu =0.5$. With increasing values of *E*, the values of the contractive path decrease slightly, and the values of the initial slope increase slightly. As mentioned before, the simulated shear force shows sudden drops. This causes a high scatter in the obtained results of the final shear force and also influences the angle of dilation, as drops in the shear force correspond with kinks in the measured vertical path, which causes scatter in the calculated values of the dilation angle $\psi$. Due to the mentioned scatter in the final shear force and $\psi$, it is not a surprise to see no dependency of these characteristics on the Young’s modulus *E*.

Figure 13c–f show the calculated values of contractive path, angle of dilation, initial slope and final shear force for $E=50$ GPa, $\mu =0.5$ and highest applied normal load $F_n=30$ kN. The Figures show the dependency of the results on the parameters $E/\sigma _{{\mathrm{max}}}$ and $\alpha$ for three different initial configurations in a similar manner as Fig. 12c till f. A similar dependency on $E/\sigma _{{\mathrm{max}}}$ and $\alpha$ can be seen for the contractive path and the initial slope. The results of the contractive path show very little scatter, while the results of the initial slope show some scatter for high $\alpha$ values of low $E/\sigma _{{\mathrm{max}}}$ values. As described before, the results for $\psi$ and the final shear force show a large scatter with respect to the initial configuration, which makes it hard to see a dependency of the results on $E/\sigma _{{\mathrm{max}}}$ and $\alpha$.

In the experiments conducted, the direct shear test followed the compression test. Therefore, the initial porosities in the shear tests (final porosities of the compression test) differ for the the different sets of material parameters, see Fig. 12f. In the compression test, these porosities could be linked to the amount of yielding: high (nearly unchanged) porosities belonged to parameter sets causing little till no yielding, i.e. almost elastic material behaviour, while lower porosities belonged to parameter sets causing a higher amount of yielding in the compression test. In the direct shear test, the initial porosity together with the maximum percentage of yielding contacts (during shearing) and the maximum percentage of sliding contacts are three important internal states. In Fig. 13g correlation plots of the percentage of yielding contacts to the initial porosity, the percentage of sliding contacts and the four characteristics are shown, for $F_n=30$ kN and $\mu =0.5$. Those parameter sets which cause only few yielding contacts have a high initial porosity. Thus, these parameters cause an almost elastic material behaviour in the compression test and, as seen in the plot, also in the direct shear test. These parameter sets cause the lowest amount of sliding contacts and low contractive paths. The initial slope scatters strongly for these parameter sets. Parameter sets, which cause a high amount of yielding contacts in the compression test, result in lower final porosities and cause also a high yielding in the direct shear test. These parameter sets result in the highest amount of sliding contacts, highest contractive path and low initial slopes. Both the final shear force and the angle of dilation show no correlation the the percentage of yielding contacts (they scatter strongly).

The influence of the coefficient of friction $\mu$ on the four characteristics can be seen in Fig. 13h. The results from the DoE with $\mu =0.4, 0.5$ are shown in box plots. Additionally, for one set of parameters, $p_2$, $\mu$ is varied from 0.25 till 0.7, shown as line plots for three different initial configurations. Increasing $\mu$ from 0.4 to 0.5, leads to a small decrease in the contractive path and to an increase of the angle of dilation $\psi$. The initial slope increases with $\mu$. The final shear force shows a slight increase in the median value, while the range of obtained results widens with increasing $\mu$. Traditionally, with an increase in $\mu$ one would expect a clear increase in the final shear force. Here, a higher amount of scatter seems to be dominating, when all parameter sets are considered. The described effects can be seen more strongly in the line plots for parameter set $p_2$, because here the range of $\mu$ is broader.

Up to now, all presented results belonged to the highest applied normal force $F_n=30$ kN. The four characteristics are shown in box plots dependent on the applied normal force in Fig. 13i. The contractive path is increasing with increasing $F_n$, which can be expected. The angle of dilation shows a slight decay with increasing $F_n$ and also the range of its values reduces. The initial slope increases moderately with an increase of $F_n$. The final shear force depends strongly on the applied level of normal force. The obtained results could be evaluated with the Mohr-Coulomb failure criterion, where a linear relation between applied normal stress and shear stress is postulated and the bulk friction angle of the material can be calculated. The bulk friction angle depends on the particle shape, specimen porosity and on the contact parameters (compare the dependency of the final shear force on the parameters in Fig. 13 and in the statistical model in the next paragraph). Therefore, a detailed analysis of the bulk friction angle is beyond the scope of this work. Being one of the model parameters, the particle-particle friction coefficient will also influence the bulk friction angle. In this work, the friction coefficient is assumed to be constant based on the results of the direct shear test. However, more detailed experimental investigation might be necessary. In [13], monotone and cyclic triaxial tests on railway ballast showed a pressure dependency of the friction coefficient. Pressure dependent coefficients of friction, as used in DEM simulations in [13, 38, 39], lead to a non-linear relationship between applied normal stress and shear stress in the direct shear test.

Statistical linear models were also fitted to the four characteristics of the direct shear test, in the same way as for the compression test. For simplicity only the values belonging to $F_n=10$ kN are discussed here, as the remaining gives similar results. In Table 4, the statistically significant predictors remaining in the model after stepwise regression are marked with “x” and the $R^{2}$ value is given as well. The model of the contractive path contains all four parameters as predictors, together with the squared contribution of $E/\sigma _{{\mathrm{max}}}$ and $\alpha$ and three interactions. With and $R^{2}$ value of 0.88 the quality of the fit is acceptable. The dilation angle $\psi$, contains all four parameters as predictors, the squared contributions of *E* and $E/\sigma _{{\mathrm{max}}}$ and four interactions. As the dilation angle is calculated at a larger shear path than the contractive path, it suffers more strongly from the high scatter in the simulation of the direct shear test. This is one reason for the low value of $R^{2}=0.48$, indicating a poor fit for the model. The initial slope shows a better fit of its model with an $R^{2}$ value of 0.72. In its predictors, all parameters are included (either simple or squared contribution). This is with the exception of *E*, which is present only in the interactions. As described before, the final shear force value shows a high scatter, which is probably the main reason for the poor fit of this model ($R^{2}=0.42$). Its predictors also contain no contribution from *E* (not even in the interactions). Due to the previously described high scatter in the results of the direct shear test, the linear model fitted to $\psi$ and $S_f$ are of low quality. On the opposite, the models fitted to the contractive path and the initial slope show an acceptable quality. As in the compression test, the statistical analysis revealed several interactions, which were not discovered before. A highly complex interplay between the parameters and their interactions can be seen.

**Table 4:** Direct shear tests: statistically significant predictors and $R^{2}$ value of simplified linear models for the characteristics: contractive path, dilation angle $\psi$, initial slope and final shear force $S_f$

	Contr. path	$\psi$	Ini. slope	$S_f$
*E*	x	x		
$E/\sigma _{{\mathrm{max}}}$	x	x	x	x
$\alpha$	x	x	x	x
$\mu$	x	x	x	x
I($E^2$)		x		
I($(E/\sigma _{{\mathrm{max}}})^2$)	x	x	x	
I($\alpha ^2$)	x		x	x
I($\mu ^2$)				
I($E \cdot E/\sigma _{{\mathrm{max}}}$)	x			
I($E \cdot \alpha$)	x		x	
I($E \cdot \mu$)		x	x	
I($(E/\sigma _{{\mathrm{max}}}) \cdot \alpha$)	x	x	x	x
I($(E/\sigma _{{\mathrm{max}}}) \cdot \mu$)		x	x	
I($\alpha \cdot \mu$)		x	x	
$R^{2}$	0.88	0.48	0.72	0.42

## Parametrisation procedure

The characteristics defined in the previous Section, will now be used for the DEM model’s parametrisation. In a first step, cost functions will be formulated for the compression and the direct shear test to be able to measure the error between simulation results and experimental data. Then, a virtual validation will be conducted, which allows an investigation of parameter ambiguity. After this preparation, the DEM model’s actual parametrisation to the experimental data will be conducted. Finally, suggestions to reduce the computational effort for parametrising DEM models using similar particle shapes will be given.

### Formulation of cost functions

**Fig. 14:**
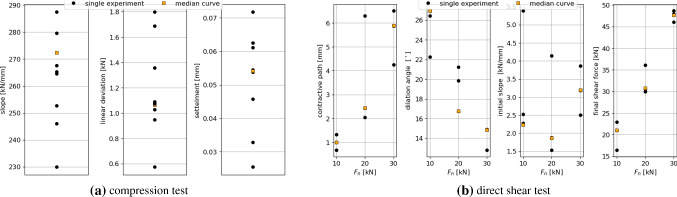
Evaluations of experimental data of Kieselkalk ballast w.r.t. characteristics for compression and direct shear test characteristics. Plotted are the characteristics of the single experiments and the characteristics of the median curve calculated in Sect. 2.1

For the comparison between simulation and experiment, the cost functions will be formulated using the characteristics introduced above, i.e. a simulation result will be considered “close” to the experiments, when its characteristics are close to those of the experiment, which is indicated by values of the cost function close (ideally equal) to zero. As the characteristics themselves have different magnitudes, it is convenient to consider relative errors. As an example, the simulation conducted with parameter set $p_i$ resulted in the compression test in a slope $sl^{\text{ sim }}(p_i)$. When the slope of the median curve of the experiments is denoted with $sl^{\text{ exp, } \text{ med }}$ and the maximal deviation of all single experiments to this value is denoted with $\delta ^{sl}$, then the relative error for the slope characteristic is defined as follows:

8 $$\begin{aligned} \epsilon ^{sl}(p_i) =\frac{|sl^{\text{ sim }}(p_i) - sl^{\text{ exp, } \text{ med }}|}{\delta ^{sl}} \end{aligned}$$

Such relative errors for the simulation conducted with parameter set $p_i$ can be formulated completely analogously for each characteristic of the compression and the direct shear test and are denoted with $\epsilon ^{ld}(p_i)$ for the linear deviation and $\epsilon ^{settle}(p_i)$ for the settlement of the compression test, $\epsilon ^{cp}(p_i)$ for the contractive path, $\epsilon ^{\psi }(p_i)$ for the dilation angle $\psi$, $\epsilon ^{iniSl}(p_i)$ for the initial slope and $\epsilon ^{S_f}(p_i)$ for the final shear force $S_f$. In Fig. 14, the measurement data of Kieselkalk ballast is evaluated for all characteristics. The characteristics of the single experiments are shown as well as the characteristics of the median curve of the experiments. This gives a good impression of the scatter of the experiments and of the values of $\delta ^{sl}, \delta ^{ld}, \ldots$ used in the formulation of the relative errors.

A cost function for the compression test can be formulated for *one simulation* using parameter set $p_i$ by using the average of the characteristics:

9 $$\begin{aligned} \epsilon ^{{odo}}_{{sim}}(p_i) =\text{ mean }\left( \epsilon ^{sl}(p_i), \,\epsilon ^{ld}(p_i), \,\ \epsilon ^{settle}(p_i) \right) \;. \end{aligned}$$

When *different initial configurations* are considered for one set of parameters, here again the average can be taken to obtain a cost function for the compression test:

10 $$\begin{aligned} \epsilon ^{{odo}}(p_i) =\text{ mean }\left( \epsilon ^{{odo}}_{{sim1}}(p_i), \, \epsilon ^{{odo}}_{{sim2}}(p_i), \, \epsilon ^{{odo}}_{{sim3}}(p_i) \right) \;. \end{aligned}$$

For the direct shear test, the cost functions for *one simulation* can be formulated analogously, for each level of applied normal force $F_n$ separately

11 $$\begin{aligned} \epsilon ^{{shear}}_{{sim}}(p_i, F_n) =\text{ mean }\Big (&\epsilon ^{cp}(p_i, F_n), \,\epsilon ^{\psi }(p_i, F_n), \nonumber \\&\epsilon ^{iniSL}(p_i, F_n) , \,\ \epsilon ^{S_f}(p_i, F_n) \Big )\;; \end{aligned}$$

and then calculating the average for all three levels of normal force:

12 $$\begin{aligned} \epsilon ^{{shear}}_{{sim}}(p_i) =\text{ mean }\Big (&\epsilon ^{{shear}}_{{sim}}(p_i, 10kN) , \, \epsilon ^{{shear}}_{{sim}}(p_i, 20kN) , \nonumber \\&\epsilon ^{{shear}}_{{sim}}(p_i, 30kN) \Big )\;. \end{aligned}$$

Again, when *different initial configurations* are considered for one set of parameters, here again the average can be taken:

13 $$\begin{aligned} \epsilon ^{{shear}}(p_i) =\text{ mean }\left( \epsilon ^{{shear}}_{{sim1}}(p_i),\, \epsilon ^{{shear}}_{{sim2}}(p_i),\, \epsilon ^{{shear}}_{{sim3}}(p_i) \right) \;. \end{aligned}$$

Finally, the cost function for simulations using parameter set $p_i$ are the average of both values for the compression and the direct shear test:

14 $$\begin{aligned} \epsilon (p_i) =\text{ mean }\left( \epsilon ^{{odo}}(p_i),\, \epsilon ^{{shear}}(p_i) \right) \;. \end{aligned}$$

The cost function $\epsilon (p_i)$ is defined as the error between simulations belonging to one parameter set $p_i$ and experimental results. Potentially, many alternative formulations can be chosen, so it is possible to use not all of the characteristics, but only some of them, or to use the **max** function instead of the **mean**. For the case considered here, the above definitions worked well.

### Virtual calibration: investigating parameter ambiguity

The actual calibration of the developed DEM model to experimental data is also called bulk material characterisation. This approach has the big problem that potentially many different parameter sets can give simulation results, which are close to the measured experimental data, [2, 6, 7]. While it can be expected that parameter sets, which are close to each other (in parameter space), can give similar simulation results, problems arise, when parameter sets not close to each other give similar results. In this case of high parameter ambiguity, it is questionable if a DEM model calibrated to one type of experiment, will be able to give reliable predictions to other experiments/applications. A virtual calibration is an ideal way to check whether a model’s parameters can be identified uniquely or if high parameter ambiguity exists. Choosing a parameter set $\hat{p}$, simulation results are calculated and used as aims for the parametrisation. In this way, the answer of the parametrisation problem is known and possible parameter ambiguities can easily be studied.

**Table 5:** Parameter values of aim curves of virtual calibration

	*E*	$E/\sigma _{{\mathrm{max}}}$	$\alpha$	$\mu$
$\hat{p_1}$	51 GPa	102	85.5$^\circ$	0.52
$\hat{p_2}$	55 GPa	102	85.5$^\circ$	0.52
$\hat{p_3}$	51 GPa	120	85.5$^\circ$	0.52
$\hat{p_4}$	51 GPa	102	84$^\circ$	0.52
$\hat{p_5}$	51 GPa	102	85.5$^\circ$	0.44
$\hat{p_6}$	51 GPa	120	84$^\circ$	0.52
$\hat{p_7}$	55 GPa	120	84$^\circ$	0.44

The virtual calibration of a DEM model is extensively studied in [2]. It is pointed out that parameter ambiguity can vary over the considered space of parameters. This means that for some points/areas in the parameter space it may be possible to parametrise the considered model uniquely, while this might not be possible for other points/areas of the parameter space. For this reason, the points chosen as virtual calibration examples are located in the area in parameter space investigated later on, see Table 5 for the exact values.

**Fig. 15:**
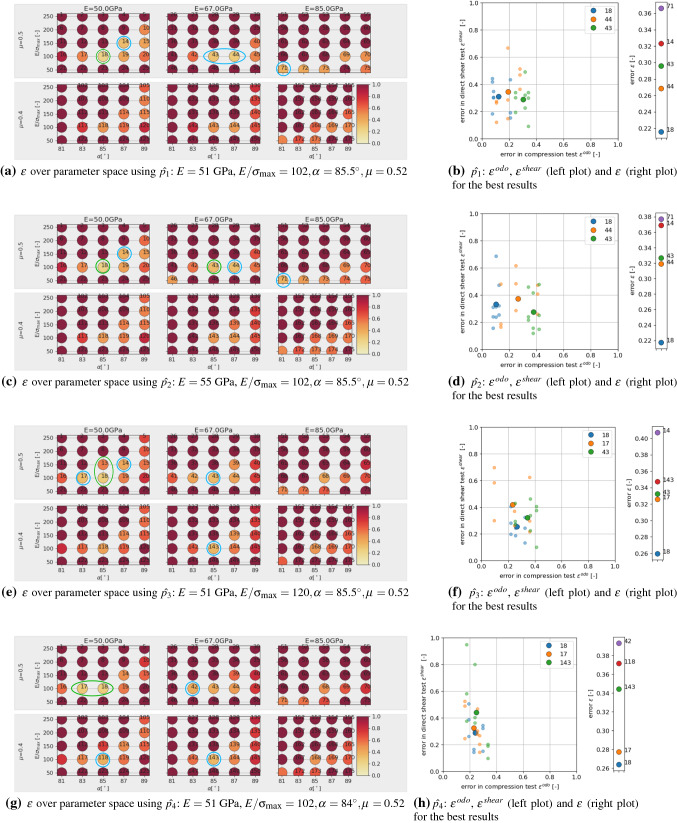
Results of virtual calibration for parameter set $\hat{p_1}, \hat{p_2}, \hat{p_3}, \hat{p_4}$

**Fig. 16:**
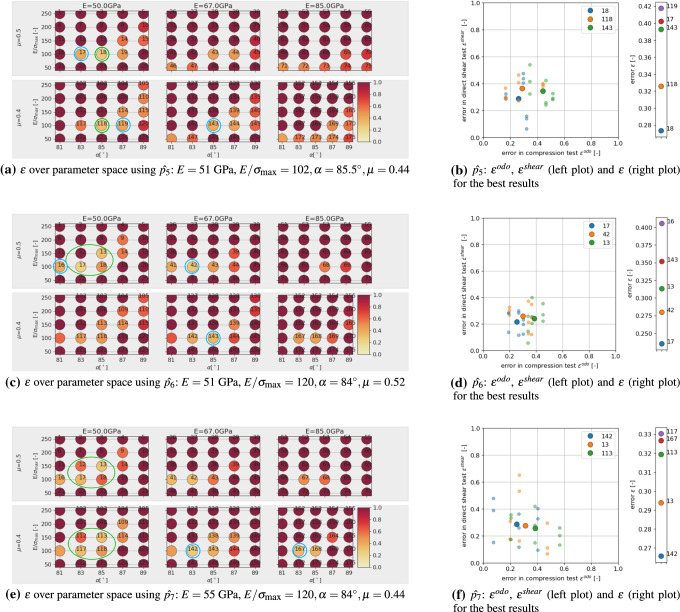
Results of virtual calibration for parameter set $\hat{p_5}, \hat{p_6}, \hat{p_7}$

From the simulations of the DoE already conducted, the cost functions $\epsilon ^{odo}$ Eq. (10), $\epsilon ^{shear}$ Eq. (13) and $\epsilon$ Eq. (14), are evaluated, where the simulation results belonging to $\hat{p_1}$ (stemming from three different initial configurations) are used instead of the experimental values. The first parameter $\hat{p_1}$ is chosen to be an easy example, this point is close to one of the points of the DoE. In Fig. 15a, the resulting values of the cost function $\epsilon$ are plotted over the parameter space. Note that the numbering of the parameter sets shown in the Figure is chosen such that $p_1$ to $p_{75}$ belong to $\mu =0.5$, while the corresponding parameter sets belonging to $\mu =0.4$ are denoted with $p_{101}$ to $p_{175}$ (in total 150 parameter sets). The point in the DoE closest to $\hat{p_1}$ is $p_{18}$ (encircled green in the Figure for improved visibility). The lowest errors w.r.t. $\epsilon$ (encircled blue in the Figure for improved visibility) are present at $p_{18}$ and some neighbouring points, but low errors also exist for higher values of *E* and even for low values of $\mu$ some moderate errors exist, always at diagonal or horizontal bands w.r.t. $\alpha , E/\sigma _{{\mathrm{max}}}$. In Fig. 15b, the five best parameter sets are shown w.r.t. the overall error $\epsilon$. The best result gives $p_{18}$, i.e. the virtual calibration succeeded to identify the parameter set closest to $\hat{p_1}$. The other parameter sets, which have low values of $\epsilon$ are neighbouring points of $p_{18}$, but also $p_{43},p_{44}$ belonging to $E=67$ GPa and $p_{71}$ belonging to $E=85$ GPa are under the five lowest values (in fact three parameter sets belonging to $E=85$ GPa are present in the ten best results). This hints at a possible higher parameter ambiguity w.r.t. parameter *E*. Also shown in Fig. 15b is a scatter plot of the three best results $p_{18}, p_{44}, p_{43}$ over $\epsilon ^{odo}$, $\epsilon ^{shear}$. Small and bright symbols correspond to single simulations belonging to the three different initial configurations; simulations of one initial configuration give one value of $\epsilon ^{odo}$ for the compression test and three values of $\epsilon ^{shear}$ belonging to the three levels of applied normal load in direct shear test. Thus, for one parameter set nine points are plotted in the Figure. For clarity, the mean value of these nine points is plotted in the Figure as well using large symbols. While in this case $p_{18}$ could be successfully identified as best result, it is clear that the scatter is a potential problem.

The points $\hat{p_2}$ to $\hat{p_5}$ are constructed to change always one parameter value from $\hat{p_1}$ and the results are presented in the same way in Fig. 15. For $\hat{p_2}$ the value of *E* is increased from 51 to 55 GPa. From Fig. 15c, d it can be seen that the resulting errors hardly change. Thus, from the parameter sets used in the DoE this change in *E* cannot be detected.

For parameter $\hat{p_3}$ the value of $E/\sigma _{{\mathrm{max}}}$ is increased from 102 to 120, therefore this parameter is now located between $p_{18}$ and $p_{13}$ (encircled green in Fig. 15e for improved visibility). The evaluation in Fig. 15e, f shows that lowest errors occur at $p_{18}$ (indicating a successful calibration), $p_{17}$ (same $E/\sigma _{{\mathrm{max}}}$ and lower $\alpha$), $p_{43}$ (higher *E*), $p_{143}$ (lower $\mu$ and higher *E*) and $p_{14}$(higher $E/\sigma _{{\mathrm{max}}}$ and higher $\alpha$) ; these values are encircled blue in the Figure for improved visibility. The errors of the single simulations in the scatter plot in Fig. 15f moved closer together and overlay with the points belonging to different parameter sets. It is notable, that increasing $E/\sigma _{{\mathrm{max}}}$ can partly be compensated by lowering $\alpha$ or $\mu$ or increasing *E* using the available simulation data from the DoE.

For parameter $\hat{p_4}$ the value of $\alpha$ is decreased from 85.5$^\circ$ to 84$^\circ$, such that this parameter is now located between $p_{18}$ and $p_{17}$. From the evaluation in Fig. 15g, h this is well recognised: the lowest errors occur at these two points. Further low errors occur at $,p_{42},p_{118},p_{143}$ and belong to higher values of *E* and/or lower $\mu$.

For parameter $\hat{p_5}$ the value of $\mu$ is decreased from 0.52 to 0.44, such that this parameter is now located between $p_{18}$ and $p_{118}$. This is well detected in the evaluation in Fig. 16a, b, as the lowest errors occur at these points. Further low errors occur at the neighbouring points $p_{17}, p_{119}$, but also at $p_{143}$ belonging to a higher value of *E*. In the scatter plot in Fig. 16b it can be seen that $p_{17}$ gives comparable results in the compression test and its higher error in $\epsilon$ stems from slightly higher errors in the direct shear test.

The next point, $\hat{p_6}$ is constructed from $\hat{p_1}$ by changing both values of $E/\sigma _{{\mathrm{max}}}$ and $\alpha$. This point is located between $p_{12}, p_{13}, p_{17}, p_{18}$. In the evaluation in Fig. 16c, d, $p_{17}$ gives the lowest error, $p_{13}$ the third smallest error, indicating a successful calibration. Further low errors occur at $p_{42}$ (higher *E* value), and $p_{143}$ (higher *E* and lower $\mu$ value) and $p_{16}$ (lower $\alpha$). Also, the errors of the single simulations in the scatter plot in Fig. 16d overlay for points belonging to the three different parameter sets.

Finally, the last point of the virtual calibration, $\hat{p_7}$, is placed in the middle of existing parameter sets of the DoE: between $p_{12}, p_{13}, p_{17}, p_{18}, p_{112}, p_{113}, p_{117}, p_{118}$ and $p_{37}, p_{38}, p_{42}, p_{43}, p_{137}, p_{138}, p_{142}, p_{143}$. In the evaluation in Fig. 16e, f, low errors occur at the mentioned parameter sets, which surround $\hat{p_7}$, and some neighbouring points. In Fig. 16f the four parameter sets with lowest error $\epsilon$, belong to the direct neighbourhood of $\hat{p_7}$, while the fifth parameter set, $p_{167}$, belong, to $E=85$ GPa (two parameter sets belonging to $E=85$ GPa are present in the ten best results), which shows again possible problems in identifying *E*. In the scatter plot in Fig. 16f, the errors of the single simulations show a higher scatter then for the other cases and again overlay for points belonging to the three different parameter sets.

In all cases of the virtual calibration, the parameter sets with the lowest error were close to the chosen aims, so that the calibration can be considered successful. However, some parameter ambiguity was observed for parameters *E* and $\mu$. All points, $\hat{p_1}$ to $\hat{p_7}$, had a value of *E* of either 51 or 55 GPa. In all cases of the virtual calibration, the parameter sets with the five lowest values of $\epsilon$ contained parameter sets belonging to $E=67$ GPa, in three cases even parameter sets belonging to $E=85$ GPa were present. For parameter $\mu$, in the cases of $\hat{p_1}, \hat{p_2}, \hat{p_7}$, parameter sets with the five lowest values of $\epsilon$ contained parameter sets belonging to $\mu =0.4$ although these points had $\mu =0.52$. Also, the scatter of the simulation results can be strong. This gives a strong hint that applying optimisation methods for the parametrisation process may not be useful: the response of the cost function $\epsilon$ will not be smooth and the optimisation method is expected to suffer from this non-smoothness. Moreover, a detailed search of parameter sets very close to each other will cause a high computational effort, but is likely bring little to no improvement of the obtained results due to the observed scatter.

### Parametrisation to experiments

**Fig. 17:**
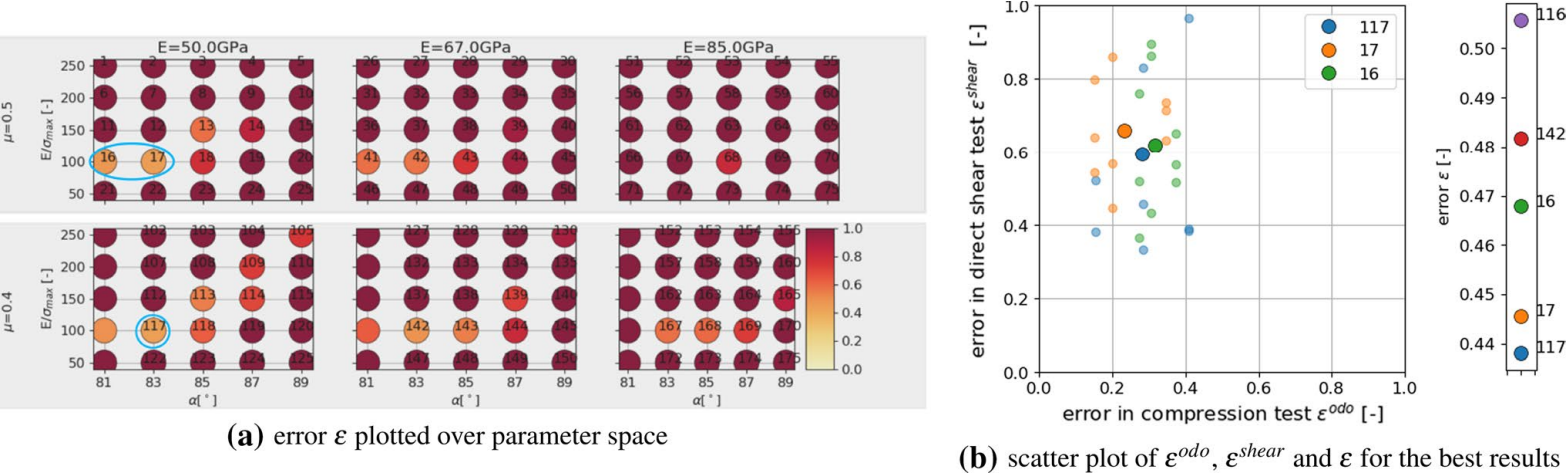
Parametrisation of DEM model with experimental data: evaluation of DoE simulations

In the next step, the DEM model will be calibrated to the experimental data. At first, the already existing simulation results of the DoE will be evaluated. Figure 17, shows the results in the same way as before for the virtual calibration. Shown are the resulting values of $\epsilon$ plotted over the parameter space in Fig. 17a. Low errors (encircled blue for improved visibility) occur for low values of *E* and both values of $\mu$. Figure 17b shows the five parameter sets with lowest values of $\epsilon$. The scatter of the results already makes it hard to decide, which is the “best” result. The four parameter sets with lowest $\epsilon$ values belong to $E=50$ GPa, $E/\sigma _{{\mathrm{max}}}=100$, $\alpha =81, 83^\circ$ and $\mu =0.4,0.5$.

**Table 6:** Parameters and their used levels in the local full DoE plan

Parameter					
*E* (GPa)	50				
$E/\sigma _{{\mathrm{max}}}(-)$	85	100	115		
$\alpha$ ($^\circ$)	80	81	82	83	84
$\mu (-)$	0.4	0.45	0.5		

**Fig. 18:**
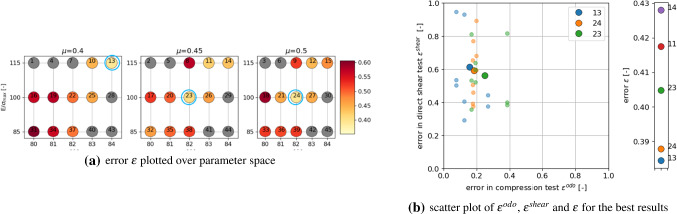
Parametrisation of DEM model with experimental data: evaluation of additional simulations (for $E=50$ GPa)

**Fig. 19:**
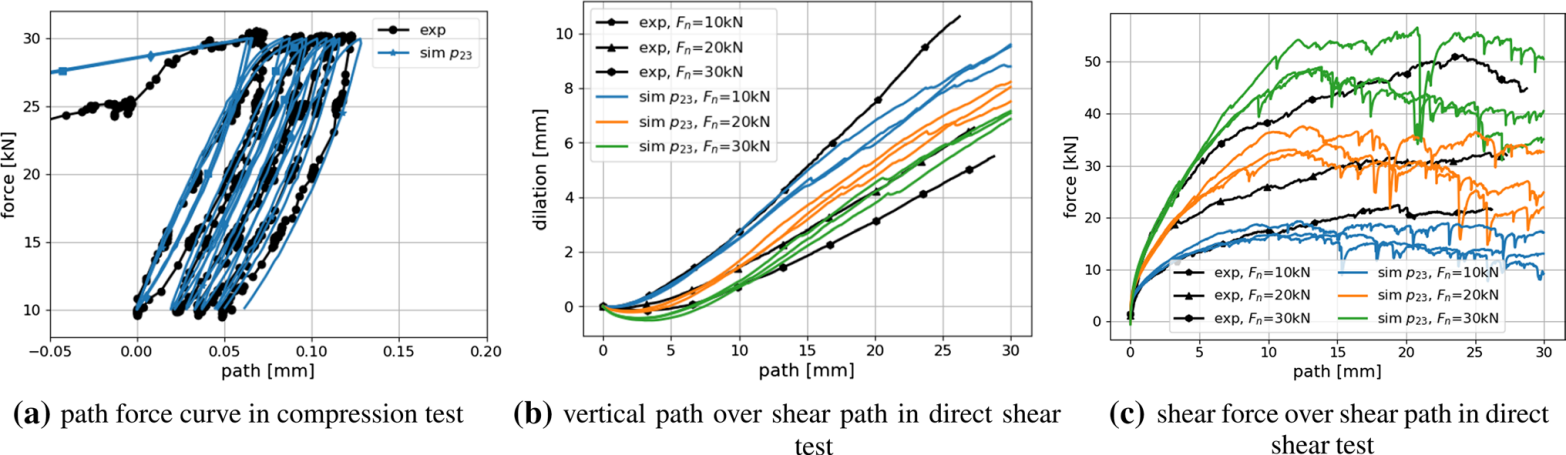
Experimental results plotted together with simulation results of $p_{23}:E=50$GPa, $E/\sigma _{{\mathrm{max}}}=100, \alpha =82^{\circ }, \mu =0.45$

With a lower amount of scatter present in the results, these four parameter sets would give good starting points for a local optimisation method, see e.g. [31] for a good overview of available methods and an efficient surrogate model based calibration. However, due to the scatter of the results and parameter ambiguity for *E* and $\mu$ this is not considered reasonable. In contrast, it is chosen to conduct additional simulations in the neighbourhood of the before mentioned parameter sets. A local full DoE is set-up with parameter levels given in Table 6. As all four parameter sets belong to $E=50$ GPa and as the virtual calibration hinted that *E* might be difficult to identify, it is decided not to vary this parameter. The other parameter levels were chosen to surround the four values. In total, the local DoE consists of 45 parameter sets. To reduce the number of simulations, the linear statistical models for slope and linear deviation in the compression test were used to predict the results. Using these predicted values, 16 parameter sets could be excluded. For the remaining parameter sets, simulations for three different initial configurations were conducted. Evaluations of the cost function $\epsilon$ are shown in Fig. 18 (low errors are encircled blue). Note that all results have the same $E=50$ GPa, the three subplots in Fig. 18a belong to different $\mu$ values; grey points belong to excluded parameter sets. Figure 18b shows the parameter sets with the five lowest vales of $\epsilon$. The three lowest values belong to three different $\mu$ values. In this local DoE, the low $\epsilon$ values are neighbouring in parameter space, but it is hard to choose one of them to be best, due to the scatter, Fig. 18b. At this point, conducting further simulations seems to makes no sense. From the parameter sets belonging to the three lowest values of $\epsilon$, $p_{23}$ with $E=50$ GPa, $E/\sigma _{{\mathrm{max}}}=100, \alpha =82^\circ$ and $\mu =0.45$ is chosen because it lies in the middle between the to other points. The good quality of the fit to the experimental data is confirmed by visual inspection of the simulation results, see Fig. 19.

### Suggestions for speeding up the process

The investigations above allowed a successful parametrisation of a DEM model for one simple particle shape. Since the influence of the parameters on the simulation results was also investigated beforehand, the overall calculation effort was very high. In this subsection, steps for a more efficient parametrisation of DEM models using simple particle shapes will formulated. To do so a different particle shape will be used; one of the 20 possible particle shapes identified in the DEM particle shape modelling paper [42]. Shape no. 9 consists of three non-overlapping spheres with the radii 9.9, 5.1 and 5.1 mm, see Fig. 10. At first, the parameter $\mu _{{\mathrm{ini}}}$ has to be chosen, such that the correct mass is filled in the box during the rainfall procedure in specimen generation. Then a DoE is defined for the parameters, based on the experience gained so far, see Table 7.

**Table 7:** Parameters and their used levels in the local full DoE plan for shape no. 9

Parameter				
*E* (GPa)	50	67		
$E/\sigma _{{\mathrm{max}}}(-)$	75	100	125	150
$\alpha$ ($^\circ$)	81	83	85	87
$\mu (-)$	0.4	0.5		

**Fig. 20:**
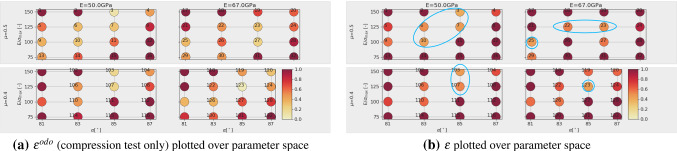
Parametrisation for shape no. 9, step 1: simulations with one initial configuration

**Fig. 21:**
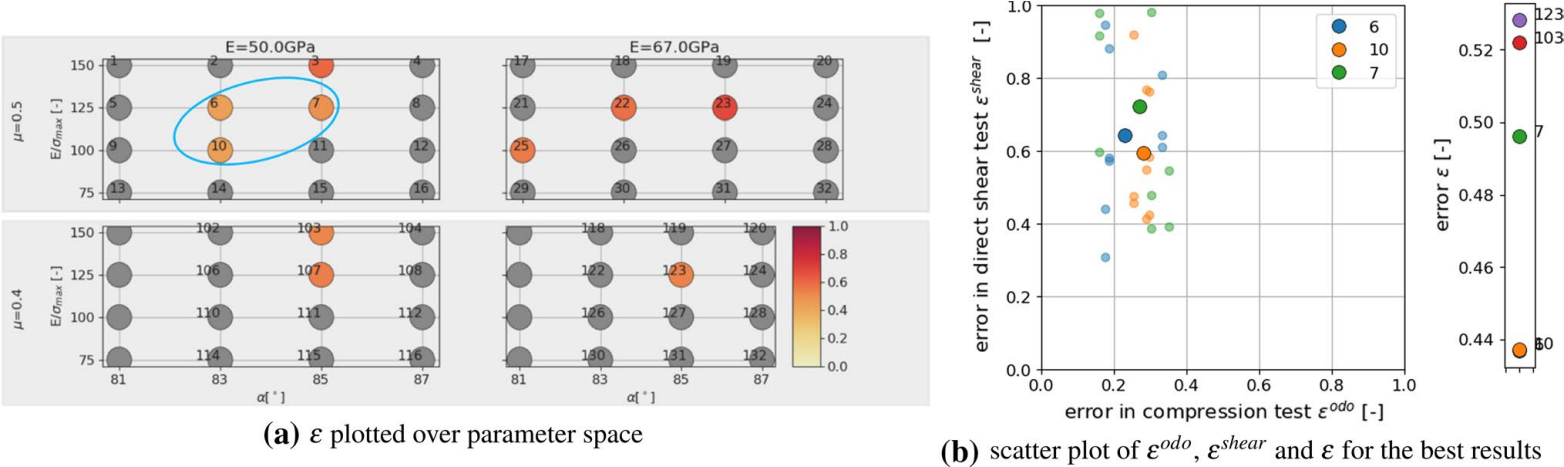
Parametrisation for shape no. 9, step 2: simulations with three initial configurations

**Fig. 22:**
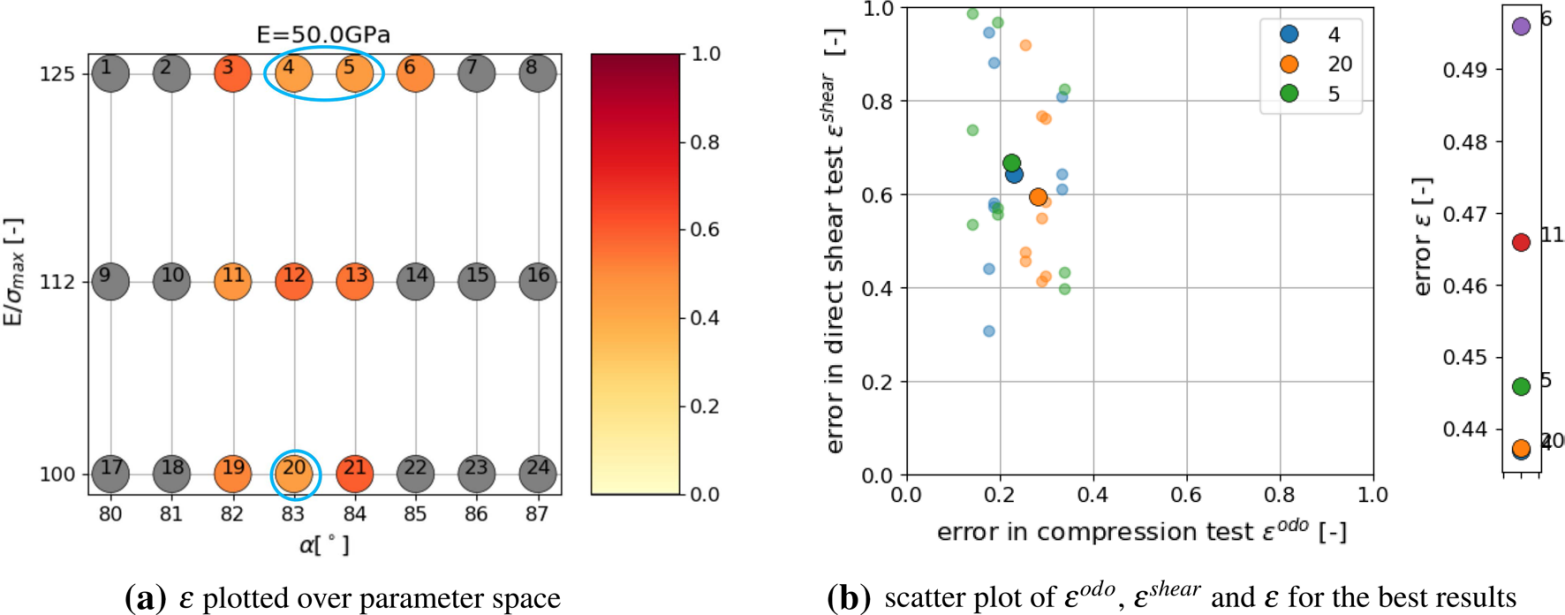
Parametrisation for shape no. 9, step 3: refined parameter space, simulations with three initial configurations

The following steps are conducted to achieve a parametrisation with reduced computational effort:

- *Step 1:* first screening of parameter space
  - simulate compression test for all parameter sets in the DoE for one initial configuration (64 runs)
  - evaluate $\epsilon ^{odo}$, see Fig. 20a
  - choose *M* parameter sets with lowest errors and simulate direct shear test
  - evaluate $\epsilon ^{shear}$ and $\epsilon$, see Fig. 20b
- *Step 2:* repetition simulations
  - choose *N* parameter sets with lowest errors
  - conduct repetition simulations with different initial configurations for compression and direct shear tests
  - evaluate $\epsilon ^{odo}$, $\epsilon ^{shear}$ and $\epsilon$, see Fig. 21a
  - investigate amount of scatter of results, see Fig. 21b
- *Step 3:* possibly simulation for additional parameter sets
  - if necessary, define additional parameter sets (possibly using statistical models for prediction)
  - conduct repetition simulations with different initial configurations for compression and direct shear tests
  - evaluate $\epsilon ^{odo}$, $\epsilon ^{shear}$ and $\epsilon$, see Fig. 22b

**Fig. 23:**
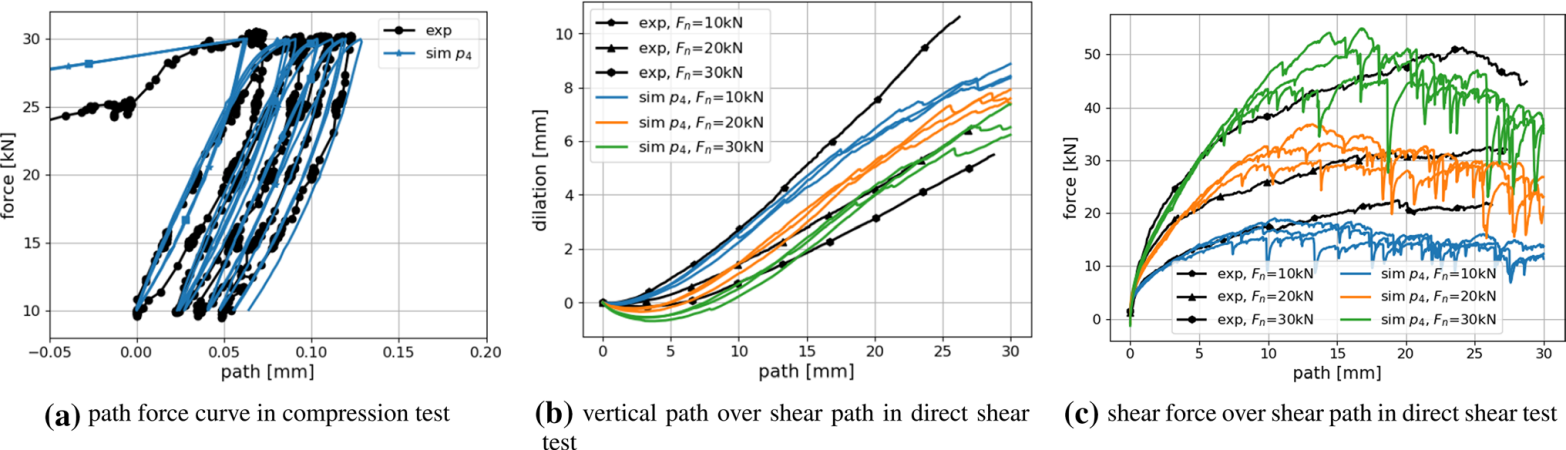
Experimental results plotted together with simulation results of $p_{4}$: $E=50$GPa, $E/\sigma _{{\mathrm{max}}}=125, \alpha =83^{\circ }, \mu =0.5$

From the simulation runs belonging to only one initial configuration, shown in Fig. 20, the 10 parameter sets with lowest error are chosen (encircled blue for improved visibility). Repetition simulations are conducted, such that for each parameter set three initial configurations are simulated, compare Fig. 21 for the evaluation results. Again the lowest errors are encircled blue: parameter sets $p_6, p_7, p_{10}$ are neighbouring, which facilitates to conduct additional simulations. For $E=50$ GPa, $\mu =0.5$, additional simulations are conducted, see Fig. 22 for the evaluation. The additional simulation do not reduce the error further; lowest errors occur at $p_{4}$ (corresponds to $p_{6}$ in Fig. 21) and $p_{20}$ (corresponds to $p_{10}$ in Fig. 21). Therefore the parametrisation process is stopped here. The best results obtained with parameter set $p_4$ can be seen in Fig. 23.

## Conclusions and outlook

This work is part of a series of papers on DEM modelling of railway ballast. Two types of ballast were tested for their bulk behaviour in compression and direct shear tests, [37]. Single stone measurements on the coefficient of friction, [36], and the Young’s modulus were conducted, [44]. Based on 3D scans of single ballast stones a shape analysis was conducted in [45]. The shape descriptors found were then used in DEM particle shape modelling to construct simple particle shapes (clumps of three spheres) with similar shape descriptors as the real ballast stones, [42]. Investigating packing behaviour, 20 possible particle shapes were identified for modelling the two considered ballast types. Particle contact modelling was addressed in [37] using simple particle shapes: while it was not possible to parametrise the Hertz-Mindlin law with compression and direct shear tests, this was achieved using the CDM law. The CDM law has four parameters, thus parametrisation of DEM models for 20 particle shapes is a huge task.

This work took the following steps: As the CDM law is not as frequently used as the linear spring or Hertz-Mindlin law, the influence of the CDM parameters on the simulation results of compression and direct shear tests were analysed in detail. Seven so-called characteristics of the simulation results of compression and direct shear tests were defined. Analysing the parameters’ influence on these characteristics, including linear statistical models, showed a highly complex interplay of the parameters and their interactions.

As the CDM law involves four parameters (instead of two parameters as Hertz-Mindlin or the linear spring law), it is very important to investigate parameter ambiguity. High parameter ambiguity means that different (non neighbouring) parameter sets give the same (or similar) simulation results. It is questionable whether a DEM model parametrised under such conditions to one type of experiment, can give reliable predictions of different experiments or applications. Therefore, virtual calibration was used in this work to check for parameter ambiguity when tying to parametrise the CDM law with the compression and direct shear tests. For all seven considered test cases, the calibration was successful, but some parameter ambiguity with respect to *E* and $\mu$ was observed.

Combining the knowledge of the the ballast single stones measurements, the influence of the parameters on the simulation results and the possible parameter ambiguity, the DEM model was parametrised to the measurement data of compression and direct shear tests. Suggestions for reducing the computational effort of this parametrisation are given and tested for a second simple particle shape. Presumably, selection of parameter ranges and step sizes have to be adapted by experience. Also, the scatter of the results will differ for different shapes and will determine, at which point a further refinement of parameters cannot be expected to improve the results.

Future work is planned on parametrising the DEM models for the particle shapes found in [42]. This could enable the investigation of the influence of particle shape on the simulation results and include validation tests.

## Data Availability

The datasets generated and analysed during the current study are openly available in the zenodo.org repository, see [35, 41, 43, 44].

## Footnotes

1 project partners: Deutsche Bahn (DB), Austrian Federal Railways (ÖBB), Swiss Federal Railways (SBB) and Schweizerische Südostbahn (SOB), Institute of Railway Engineering and Transport Economy: Graz University of Technology

## References

[1] Ahmed S, Harkness J, Le Pen L, Powrie W, Zervos A (2016). Numerical modelling of railway ballast at the particle scale. Int. J. Numer. Anal. Methods Geomech..

[2] Ben Turkia S, Wilke DN, Pizette P, Govender N, Abriak NE (2019). Benefits of virtual calibration for discrete element parameter estimation from bulk experiments. Granul. Matter.

[3] Berghold, A.: Wirkungsweise des schotters im gleis unter verschiedenen randbedingungen. Ph.D. thesis, Graz University of Technology (2016)

[4] Berghold A (2016). Wirkungsweise von unterschiedlichen Gleisschotterarten mit und ohne Schwellenbesohlung. ZEVrail.

[5] Chen C, Indraratna B, McDowell G, Rujikiatkamjorn C (2015). Discrete element modelling of lateral displacement of a granular assembly under cyclic loading. Comput. Geotech..

[6] Coetzee C (2016). Calibration of the discrete element method and the effect of particle shape. Powder Technol..

[7] Coetzee C (2017). Review: calibration of the discrete element method. Powder Technol..

[8] Cundall PA, Strack ODL (1979). A discrete numerical model for granular assemblies. Geotechnique.

[9] de Bono J, Li H, McDowell G (2020). A new abrasive wear model for railway ballast. Soils Found..

[10] Ferellec JF, McDowell G (2010). Modelling realistic shape and particle inertia in DEM. Geotechnique.

[11] Ferellec JF, McDowell GR (2010). A method to model realistic particle shape and inertia in DEM. Granul. Matter.

[12] Gao R, Du X, Zeng Y, Li Y, Yan J (2012). A new method to simulate irregular particles by discrete element method. J Rock Mech Geotech Eng.

[13] Harkness J, Zervos A, Le Pen L, Aingaran S, Powrie W (2016). Discrete element simulation of railway ballast: modelling cell pressure effects in triaxial tests. Granul Matter.

[14] Hoang T, Alart P, Dureisseix D, Saussine G (2011). A domain decomposition method for granular dynamics using discrete elements and application to railway ballast. Ann. Solid Struct. Mech..

[15] Huang H, Tutumluer E (2011). Discrete element modeling for fouled railroad ballast. Constr. Build. Mater..

[16] Huang H, Tutumluer E (2014). Image-aided element shape generation method in discrete-element modeling for railroad ballast. J. Mater. Civ. Eng..

[17] Indraratna B, Ngo N, Rujikiatkamjorn C, Vinod J (2014). Behavior of fresh and fouled railway ballast subjected to direct shear testing: discrete element simulation. Int. J. Geomech..

[18] Indraratna B, Thakur P, Vinod J (2010). Experimental and numerical study of railway ballast behavior under cyclic loading. Int. J. Geomech..

[19] Irazabal J, Salazar F, Onate E (2017). Numerical modelling of granular materials with spherical discrete particles and the bounded rolling friction model application to railway ballast. Comput. Geotech..

[20] Kumar N, Suhr B, Marschnig S, Dietmaier P, Marte C, Six K (2019). Micro-mechanical investigation of railway ballast behavior under cyclic loading in a box test using DEM: effects of elastic layers and ballast types. Granul. Matter.

[21] Laryea S, Baghsorkhi MS, Ferellec JF, McDowell G, Chen C (2014). Comparison of performance of concrete and steel sleepers using experimental and discrete element methods. Transport. Geotech..

[22] Lu M, McDowell G (2010). Discrete element modelling of railway ballast under monotonic and cyclic triaxial loading. Geotechnique.

[23] Miao CX, Zheng JJ, Zhang RJ, Cui L (2017). DEM modeling of pullout behavior of geogrid reinforced ballast: the effect of particle shape. Comput. Geotech..

[24] Mortensen J, Faurholt JF, Hovad E, Walther JH (2021). Discrete element modelling of track ballast capturing the true shape of ballast stones. Powder Technol.

[25] Ngo NT, Indraratna B (2016). Improved performance of rail track substructure using synthetic inclusions: experimental and numerical investigations. Int. J. Geosynth. Ground Eng..

[26] Oliver W, Pharr G (1992). An improved technique for determining hardness and elastic modulus using load and displacement sensing indentation experiments. J. Mater. Res..

[27] Ouhbi N, Voivret C, Perrin G, Roux JN (2017). 3d particle shape modelling and optimization through proper orthogonal decomposition. Granul. Matter.

[28] Qian Y, Lee SJ, Tutumluer E, Hashash YMA, Ghaboussi J (2018). Role of initial particle arrangement in ballast mechanical behavior. Int. J. Geomech..

[29] Qian Y, Mishra D, Tutumluer E, Kazmee HA (2015). Characterization of geogrid reinforced ballast behavior at different levels of degradation through triaxial shear strength test and discrete element modeling. Geotext. Geomembr..

[30] R Core Team: R: A Language and Environment for Statistical Computing. R Foundation for Statistical Computing, Vienna, Austria (2016). https://www.R-project.org/

[31] Richter C, Roessler T, Kunze G, Katterfeld A, Will F (2020). Development of a standard calibration procedure for the DEM parameters of cohesionless bulk materials—part II: efficient optimization-based calibration. Powder Technol..

[32] Roessler T, Richter C, Katterfeld A, Will F (2019). Development of a standard calibration procedure for the DEM parameters of cohesionless bulk materials—part I: solving the problem of ambiguous parameter combinations. Powder Technol..

[33] Roth LK, Jaeger HM (2016). Optimizing packing fraction in granular media composed of overlapping spheres. Soft Matter.

[34] Stahl M, Konietzky H (2011). Discrete element simulation of ballast and gravel under special consideration of grain-shape, grain-size and relative density. Granul. Matter.

[35] Suhr B, Butcher TA, Lewis R, Six K (2020). Cyclic friction tests of ballast stones interfaces under varying vertical load [data set]. Zenodo.

[36] Suhr B, Butcher TA, Lewis R, Six K (2020). Friction and wear in railway ballast stone interfaces. Tribol. Int..

[37] Suhr B, Marschnig S, Six K (2018). Comparison of two different types of railway ballast in compression and direct shear tests: experimental results and DEM model validation. Granul. Matter.

[38] Suhr B, Six K (2016). Friction phenomena and their impact on the shear behaviour of granular material. Comput. Part. Mech..

[39] Suhr B, Six K (2016). On the effect of stress dependent interparticle friction in direct shear tests. Powder Technol..

[40] Suhr B, Six K (2017). Parametrisation of a DEM model for railway ballast under different load cases. Granul. Matter.

[41] Suhr B, Six K (2018). Compression tests and direct shear test of two types of railway ballast [data set]. Zenodo.

[42] Suhr B, Six K (2020). Simple particle shapes for DEM simulations of railway ballast—influence of shape descriptors on packing behaviour. Granul. Matter.

[43] Suhr B, Six K, Skipper W, Lewis R (2020). 3D scans of two types of railway ballast including shape analysis information [data set]. Zenodo.

[44] Suhr B, Six K, Skipper WA, Lewis R (2021). Young’s modulus of railway ballast stones measured via nano-indentation [data set]. Zenodo.

[45] Suhr B, Skipper WA, Lewis R, Six K (2020). Shape analysis of railway ballast stones: curvature-based calculation of particle angularity. Sci. Rep..

[46] Thornton C, Yin K (1991). Impact of elastic spheres with and without adhesion. Powder Technol..

[47] Tutumluer E, Qian Y, Hashash YM, Ghaboussi J, Davis DD (2013). Discrete element modelling of ballasted track deformation behaviour. Int. J. Rail Transport..

[48] Šmilauer, V., et al.: Yade Documentation 2nd ed. The Yade Project (2015). 10.5281/zenodo.34073. http://yade-dem.org/doc/

